# A Bibliometric Analysis of Research Publications of the Bucharest University of Economic Studies in Time of Pandemics: Implications for Teachers’ Professional Publishing Activity

**DOI:** 10.3390/ijerph19148779

**Published:** 2022-07-19

**Authors:** Adriana Ana Maria Davidescu, Margareta-Stela Florescu, Liviu Cosmin Mosora, Mihaela Hrisanta Mosora, Eduard Mihai Manta

**Affiliations:** 1 Department of Statistics and Econometrics, The Bucharest University of Economic Studies, 010552 Bucharest, Romania; 2 Department of Education, Training and Labour Market, National Scientific Research Institute for Labour and Social Protection, 010643 Bucharest, Romania; 3 Department of Administration and Public Management, Bucharest University of Economic Studies, 010374 Bucharest, Romania; margareta.florescu@ari.ase.ro; 4 Department of Economics and Economic Policies, Bucharest University of Economic Studies, 010374 Bucharest, Romania; comin.mosora@economie.ase.ro (L.C.M.); mihaela.mosora@economie.ase.ro (M.H.M.); 5 Doctoral School of Cybernetics and Statistics, Bucharest University of Economic Studies, 010374 Bucharest, Romania; eduard.manta@csie.ase.ro

**Keywords:** bibliometric analysis, Bucharest University of Economic Studies, Web of Science, Scopus, research publications, pandemics

## Abstract

The epidemic has forced the academic world, regardless of nation of origin, to unify to find a response to the economic and social difficulties we confront as quickly as possible. This paper investigates how academic performance in terms of scientific publications, especially during the pandemic period, may constitute the premises for boosting professional well-being. The analysis focuses on the researchers and professors of the Bucharest Academy of Economic Studies, analysing in a comparative way the academic performance during the pandemic as a fundamental side of their professional career. To do that, two samples of scientific publications collected between January 2020 and December 2021 were investigated. The first sample comprised 1411 documents indexed in the WoS database, while the second one was formed by 876 documents indexed in the Scopus database. All samples were published during the pandemic and have the university’s affiliation. The empirical findings indicated that the pandemic has created a boost in the number and quality of medical publications for the professors at the Bucharest University of Economic Studies. They created new multidisciplinary teams (economics and medicine), strengthening and widening national and international collaborations.

## 1. Introduction

COVID-19 is the subject of high-level, widespread debate generated by its vast social and economic global impact. Everybody is talking about the SARS-CoV-2 syndrome and how it has radically affected our lives, social relationships, workplace activity, and interactions.

Researchers in virtually all fields are trying to find an answer, a solution to this pandemic, while, at the same time, attempting to anticipate its economic and social impact at the global level. Most scientific research papers published after 2020 pertain to the SARS-CoV-2 syndrome, and this is also the case in Romania, where the topic has been the subject of rather extensive scientific research and literature. This paper brings an element of novelty by endeavouring to summarise the scientific papers published by the research professors from the Bucharest University of Economic Studies through a bibliometric analysis.

The topic selected is highly relevant in the current context, because we can use it to identify the main future research areas in the economic field and the various angles from which Romanian researchers approached this topic. The SARS-CoV-2 syndrome will undoubtedly become the catalyst for several changes in this academic research field at the global level.

The study aims to explore the Bucharest University of Economic Studies research publications, emphasizing the trends of the literature and future potential research paths using science mapping, which permits the assessment of scientific information. We collected articles from the Web of Science and Scopus databases from the pandemic era of 2020–2021, researching a total of 1411 documents indexed in WoS and 876 documents indexed in Scopus and focused on author, keyword, paper, journal, and subject analysis to elaborate on the temporal growth terms of publications.

This paper aims to provide answers to the following research questions: RQ1. Who are the top researchers in pandemics, and what are the significant journals? RQ2. Is there a geographic concentration of research, and how is the research interconnected? RQ3. What are the top keywords and notable research clusters? RQ4. How is the research progressing in the field during the COVID-19 outbreak? RQ5. What is the intellectual, social, and conceptual framework of the primary research publications?

Another important question that the research aims to provide a response to is: What is to be gained by searching both databases? Are there significant results in comparatively investigating both databases?

This paper is divided into ten sections. The opening section is devoted to briefly demonstrating the topic’s importance. The following two sections are devoted to theoretical ideas and the most significant works on the topic. The fourth section is devoted to data presentation and working methods, while the following sections display empirical results, including descriptive statistics; cluster analysis; and conceptual, intellectual, and social structures for both analyses (WoS and Scopus). The key conclusions are presented at the end of the study.

## 2. Literature Review

As of the beginning of the year 2000, we saw two outbreaks at the global level: severe acute respiratory syndrome (SARS) and the Middle East respiratory syndrome (MERS). The new coronavirus strain (SARS-CoV-2) outbreak resulted in the World Health Organization (WHO) declaring a pandemic on 11 March 2020. The SARS-CoV-2 syndrome resulted in the deaths of over 5 million persons, a much higher number than the statistics for the other two outbreaks.

The pandemic caused by the new virus severely upset the global economy and contributed to numerous economic and social losses. International institutions declared that the world economy is in a recession that may be more severe than the 2009 one [1,2].

The new pandemic significantly impacted the academic community and promptly generated extensive research. Odone et al. concluded that there has never been such a large volume of scientific works on a single topic [3]. Over 10,000 scientific articles on the SARS-CoV-2 syndrome were published during the 20 January–7 May 2020 period, and 33.33% of the published works focused on clinical management. The United States, China, and Italy had significant contributions in this respect [3,4]. Hu et al. considered that interest in this topic has grown considerably, especially after the first year from the beginning of the outbreak [5].

The problems related to COVID-19 have also been analysed through bibliometric analyses. The current studies are focused on: a comparative approach between what was published between countries on this topic [6]; the most cited published works on the SARS-CoV-2 syndrome [7]; and in specific fields such as medicine [8], economics [9], and business management [10]. According to the Scopus database, the most prominent universities at the forefront of research on the SARS-CoV-2 syndrome are Huazhong University of Science and Technology, Wuhan University, and Hong Kong [11]. In the WoS database, there is a significant domination of the USA and the UK in publications [9]. Concerning the number of publications, the most influential researchers on the analysis of the SARS-CoV-2 syndrome are Huang, C., Zhu, N., and Chan, J.F. [12]. Moreover, this pandemic generated enhanced international scientific collaborations, especially among the most affected countries (the US, China, the UK, Italy, and Belgium) and among the countries with a lower GDP per capita [9,13,14,15].

The research landscape on the SARS-CoV-2 syndrome is undoubtedly the most prolific in the health sciences. Aristovnik has shown, under a bibliometric analysis, that there are four strongly intertwined scientific disciplines on this subject, namely: health sciences, life sciences, physical sciences, and social sciences and humanities [11]. Aristovnik identified the frequently used words in the literature that focused on the four disciplines [11]. Thus, for health sciences, we have “patient”, “health”, and “healthcare”; for life sciences, we have “protein”, “human”, and “vaccine”; for physical sciences, we have “factor”, “lockdown”, and “area”; and for life sciences, we have “factor”, “lockdown”, and “area”.

Mahi et al. used bibliometric methodology to analyse all the articles on economics published on the Web of Science (WoS) between 1974 and 2020 related to pandemics and epidemics [9]. The high-frequency keywords in the analysed articles were impact, epidemic, infection, cost, and efficiency. This reflects the strong connection between epidemic/pandemic and economic factors. All epidemics, including the COVID-19 pandemic, have a strong impact on the global economy. The “United States” is one of the top keywords, which indicates the geographical area where the relevant studies are concentrated.

The COVID-19 pandemic will significantly impact academic research, both short term and in the long run. Verma et al. conducted a bibliometric analysis of the literature about COVID-19 and identified four main research themes and 18 subthemes. The four themes refer to the service industry’s business, technology, and supply chain management [10].

In Romania, the crisis caused by the SARS-CoV-2 syndrome was felt primarily in the healthcare domain and strongly affected the social, economic, education, culture, and sports areas. The analysis of the research articles published on Web of Science that pertain to the COVID-19 pandemic reflects that a 27.27% focus on economics, 21.59% on business, and 21.59% on Green Sustainable Science and Technology. Additionally, over half of the articles published on economics were authored by researchers from the Bucharest University of Economic Studies. The research focused on themes that showed the impact of SARS-CoV-2 on the labour force market, the financial markets, globalisation, economic growth, tourism, air travel, and education. The research generally concentrated on the activity sectors severely affected by this pandemic.

Ahmed investigated the research contributions of Al-Jouf University, Saudi Arabia, regarding publication output in the period 2006–2017 found on the Scopus database [16]. According to the article, Al-Jouf University develops and improves research publishing production. The same idea was also developed by Maharana in 2013, researching the output of Sambalpur University for the period 2007–2011, conducting the analysis describing the growth, impact, and contribution of research carried out by the faculty members, researchers, and students [17].

Xianli et al. conducted a bibliometric analysis to identify the characteristics of the articles published in the *Journal of Economic Computation and Economic Cybernetics Studies and Research* from 1969 to 2020 [18]. The elements analysed included: the number of citations per article; the positions of the authors cited (Stoica was the first author 21 times, the second author 8 times, and the third author 3 times); the number of authors per article; the top prolific countries in terms of publication in ECECSR (with Romania at the top of the list, followed by China in second place and Iran in third place); the main topics of the articles published (“economics”, “mathematics”, and “computer”); and the highest frequency keywords in scientific publications: model, performance, prediction, selection, cointegration, and risk. An overview of the most relevant studies of research publications in a university-based approach is presented in Table 1.

Researchers used the bibliometric analysis from a different perspective in recent decades, such as analysing a specific subject, journal, or university. Therefore, conducting a bibliometric analysis on research publications of the Bucharest University of Economic Studies in a time of pandemics will highlight the impact of COVID-19 in a “new normal” way of researching.

## 3. Materials and Methods

### 3.1. Data and Methodology

The application of quantitative procedures (bibliometric analysis–citation analysis) to bibliometric data (citation and units of publication) is referred to as bibliometric methodology [27,28]. The first mention of bibliometrics dates from the 1950s [29], implying that the approach is not new. The rise of bibliometrics in the disciplines of “business, management, and accounting”; “economics, econometrics, and finance”; and “social sciences” in Web of Science and Scopus using “bibliom*” as a keyword in the “article title, abstract, and keywords” shows that it is still relatively new.

Bibliometrics-based publications have increased over time, with an average of 1021 in the previous decade, which may be ascribed to the rise of scientific research. On the other hand, classic review approaches have become onerous and unfeasible due to enormous bibliographic datasets [30]. It is worth noting that the advent of scientific databases like Scopus and Web of Science has made obtaining large volumes of bibliometric data relatively simple, and bibliometric software like Gephi, Leximancer, and VOS viewer allows for the efficient analysis of such data, resulting in a recent surge in scholarly interest in a bibliometric analysis.

Indeed, bibliometric approaches have been highlighted in a variety of domains and fields such as business [31], e-commerce [31], finance [32], management [33], marketing [34], and human resources [35]. The bibliometric analysis papers range from studying publications to collecting patterns and exploring the research field’s conceptual, intellectual, or social structure. There are several bibliometric approaches-based papers analysing research publications of universities during a specific period [31].

In order to explore the papers published with at least one author affiliated with the Bucharest University of Economic Studies in the pandemic period 2020–2021, the Web of Science (WoS) and Scopus platforms were used to extract the data. The motivation behind choosing the WOs platform for the research undertaken was primarily aimed at the research of excellence that can be quantified by ISI-indexed articles with an impact factor or AIS and which represent criteria for promotion within the Academy of Economic Studies. Additionally, the Scopus database allows us a more comprehensive image of the research field, considering the articles indexed in international databases or ISI-indexed conferences. From a professional perspective, our aim was to investigate how the pandemic period allows the Bucharest University of Economic Studies professors to publish more on topics related more to economics and medicine. The search language for this research was English. The affiliation filter of the search query used terms such as “The Bucharest University of Economic Studies”, “Bucharest University of Economic Studies”, and “Academia de Studii Economice” as keywords. Therefore, 1411 (WoS) and 876 (Scopus) documents were found across the search and were selected further in the analysis.

Analysing the number of papers published in both databases pre-pandemic (2018–2019) and during the pandemic (2020–2021) for the professors of BUES, it can be mentioned that the pandemic created a boost in the number of papers published in the field of medicine and pharmacology. Overall, there is a number of 1851 published papers indexed in WoS during 2018–2019 and 1411 papers published in 2020–2021. Regarding the Scopus database, there are 880 papers published during 2018–2019 and 876 papers published during the pandemic period. However, on medicine and pharmacology, on the WoS platform, the number of indexed articles increased from 15 papers in pre-pandemic to 26 articles during pandemic. In the case of Scopus database, the increase is even bigger, from 21 articles published in pre-pandemics to almost 60 articles during pandemics. Therefore, the pandemic has created this boost in the number and quality of medical publications.

The assessment of research is one of the many applications for bibliometric approaches, which are applied in a wide range of other sectors as well. Most bibliometric studies use two databases as their primary sources of information: Elsevier’s Scopus and Thomson Reuters’ Web of Science (WoS). There are various studies providing arguments in analysing the journal coverage of those two databases in terms of field, publishing country and language ([36,37]).

Mongeon and Paul-H [36] compared the coverage of active scholarly journals in WoS (13,605 journals) and Scopus (20,346 journals) with Ulrich’s extensive periodical directory (63,013 journals) stipulating that the use of either WoS or Scopus may introduce biases that favor Natural Sciences and Engineering as well as Biomedical Research to the detriment of Social Sciences and Arts and Humanities and English-language journals are overrepresented to the detriment of other languages. The results of Vera-Baceta et al. [38] supported the same conclusion. Mongeon and Paul-Hus [36] highlighted that the coverage of both databases differs substantially.

Empirical evidence has been brought also by the study of López-Illescas et al. [37] which compared both databases in terms of journal coverage in the field of oncology, proving that all WOS oncology journals are indexed in Scopus, but Scopus covers many more journals with much lower impact factors than WoS. Also, they were able to prove that in oncology the WoS is a genuine subset of Scopus and tends to cover the best journals from it in terms of citation impact per paper.

Distinguishing between the social sciences and humanities (SSH) and the natural sciences and engineering (NSE) subject areas, Archambault et al. [39] examines the impact of linguistic coverage of databases, showing that there is a 20 to 25% overrepresentation of English-language journals in Thomson Scientific’s databases compared to the list of journals presented in Ulrich.

Web of Science was noted as being the most selective, whereas Dimensions was identified as being the most thorough, according to Singh et al. [40]. The percentage of journals that are also indexed in Scopus and Dimensions, respectively, is around 99.11 percent and 96.61 percent of those that are indexed in Web of Science. Dimensions provides coverage for 96.42 percent of the indexed journals that are available through Scopus. The coverage of journals in the Dimensions database is the most comprehensive of any other database, with 82.22 percent more journals than Web of Science and 48.17 percent more journals than Scopus.

Into an extended perspective, investigating journal coverage via citations using different platforms, it has been proved that Google Scholar found 88% of all citations and on the second place is occupied by Microsoft Academic followed by WoS and Scopus. Scopus and WoS are not the only options available; Microsoft Academic and Dimensions offer comparable levels of coverage for a wide variety of topic areas (Martín-Martín et al. [41]).

Vieira and Gomes [42] conducted a study in which they compared ISI Web of Knowledge and Scopus from the perspective of journal coverage. They used a group of Portuguese universities as their research sample and found that approximately two thirds of the documents referenced in either of the two databases can be found in both databases, while a small fraction of one third are only referenced in one database or the other.

Analysing now the scientific production of teachers at the Bucharest University of Economic Studies during the pandemic period (2020–2021), it can highlight that, during the pandemic, a total number of 264 journals WoS-indexed were accessed by the professors at the Bucharest University of Economic Studies as the destination of their research works, with 83 of them being indexed only in WoS. On the other side, a total number of 240 Scopus-indexed journals were selected by the Bucharest University of Economic Studies professors, with 58 of them only being indexed in Scopus.

Comparing now the coverage of each database in the other, we can mention that 78.03% of WoS is covered in the Scopus database, while a much smaller proportion of 59.46% of Scopus is covered in the WoS database.

**Figure 1:**
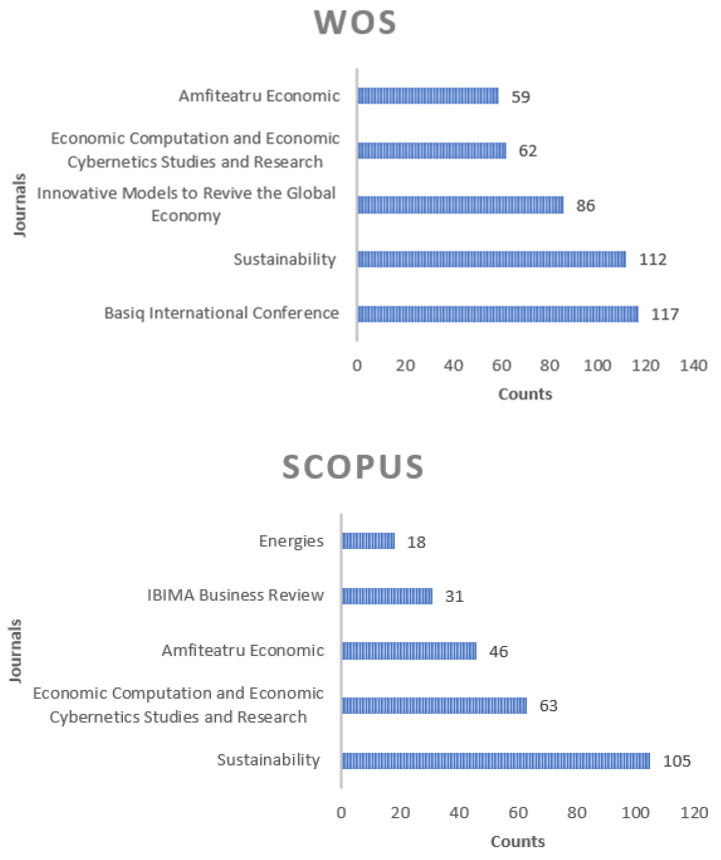
Top journals in both WoS and Scopus by the number of published papers.

Analysing comparatively top journals in both databases through the total number of published papers, it can be mentioned that Sustainability occupied the first place, followed by Economic Computation and Economic Cybernetics Studies and Research and Amfiteatru Economic, even if the ranks are a little bit different (Figure 1).

**Figure 2:**
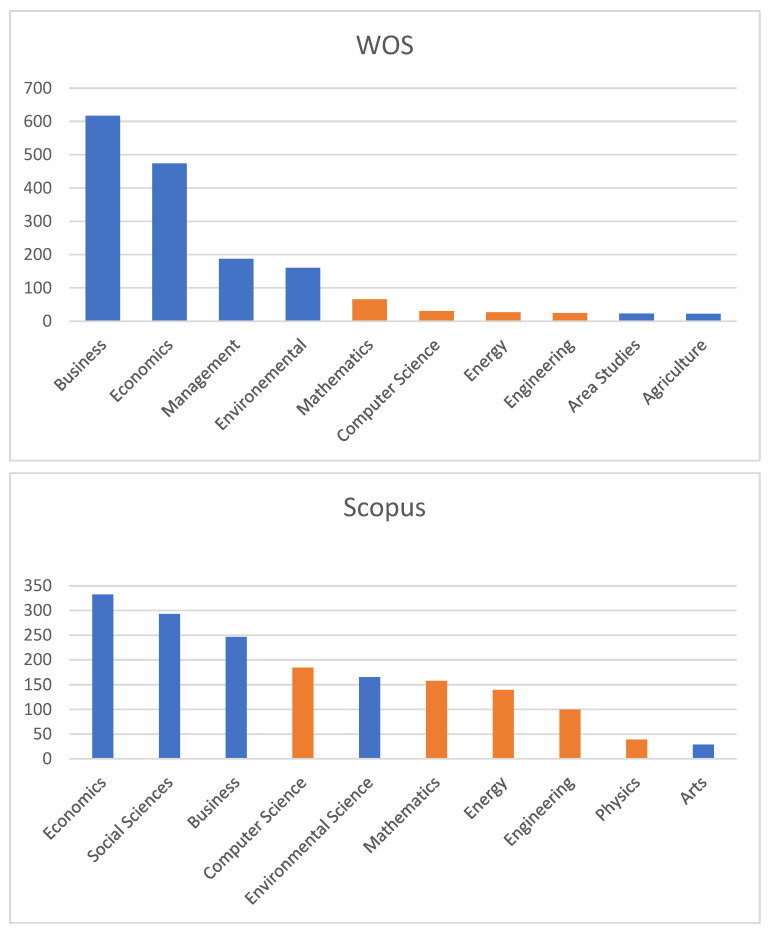
Distribution of journals in both databases by subject areas.

Analysing now the distribution of journals in both databases: Scopus and WoS, by the subject areas, it can be observed that most of the journals indexed in WoS are in the categories of Business and Economics, while, in Scopus, we encountered mostly journals from Economics and Social Sciences (Figure 2).

From the language perspective, in both databases, an overwhelming proportion of publications are written in English (only one paper was in the French language).

Based on a bibliometric analysis, we developed comparative research, investigating also the differences between both databases in terms of results. The bibliometric analysis uses the open-source packages Bibliometrix and Biblioshiny, which are run in the R language environment. Bibliometrix allows for the complete analysis and data processing of scientific publications. Biblioshiny is an online data analysis platform that encapsulates the basic Bibliometrix algorithm [43]. Biblioshiny is a web-based application that allows users to do relevant bibliometric and visual analyses.

The research methodology has been structured in three main steps: study design and data collection, analysis and visualization, and interpretation. It uses the Bibliometrix and Biblioshiny packages to display bibliometric indicators regarding publications with the authors affiliated with The Bucharest University of Economic Studies during the COVID-19 pandemic period.

The methodology is focused on a descriptive bibliometric analysis exploring the growth of publications in the field, the most abundant sources, the most cited documents, the most productive countries, author impact, the relationship between keywords–author journals, and word growth.

The study then examines the scientific publications research area’s conceptual, intellectual, and social structure from two perspectives, employing the network and cluster analyses. A network analysis based on the keyword co-occurrence and multiple correspondence analysis demonstrates the essential research themes in the conceptual framework. Thus, the research also considered the analysis of keywords and co-occurrences of words to map the structure of existing knowledge. This analysis is a beneficial systematic method in the scientific discovery of the links between the research sub-domains, monitoring and analysing the phenomenon [44] and constructing a semantic map of the domain [33]. A co-word analysis allows the use of the actual content of a plain text to capture concomitant interactions in the construction of the general framework [44]; therefore, it allows the extraction of derived scientific maps based on the words with the highest frequencies that appear in the text. Using the appropriate keyword matching algorithm (multiple correspondence analysis or MCA) provides access to the existing research’s conceptual structure and thematic map.

The co-citation network (authors, papers, and journals) is explored in the intellectual structure, while the collaboration network among authors, institutions, and nations is explored in the social structure. World clouds for abstracts, authors, and articles are clustered by coupling in a cluster analysis. A citation analysis is applied to measure the impact of the authors and publications, as it is the most conventional measure to assess the quality and scientific impact [45].

Additionally, in the research, the essential journals are determined by Bradford’s Law, which registers the journals ascending, with the highest frequency of publications being classified as the “core area”. This method is often used to understand how the literature on a particular topic is disseminated or distributed in journals and is used as a guide to determine the number of core journals on a particular topic [46]. In addition, we use the number of publications and citation information to find out the most influential country and institutions and view the geographical and institutional leadership of the research. A citation analysis may be performed by obtaining descriptive and network data.

Bibliographic coupling, co-citation, and co-word are the three standard citation analysis methodologies. If two records cite the same publication, we may claim bibliographic coupling [47], whereas co-citation [48] gauges the most citing reports. The cognitive structure of the network is mapped over time using the cooccurrence of terms in the abstract, title, or keywords in the publications [49].

The chronological growth of a scientific topic in which conceptual structures are generated through textual discourse is induced by co-word citation. In addition to a descriptive analysis, the study performs a bibliometric coupling analysis to assess the journal’s subject structure. Texts that quote the same third document, according to Kessler [47], create a bibliographic pair that addresses linked intellectual topics [50].

### 3.2. Descriptive Analysis

According to Table 2, the researchers used a sample of documents summing 1411 documents indexed in the WoS platform and 876 documents indexed in the Scopus database that had at least one author with the Bucharest University of Economic Studies affiliation. The sets of documents were found across six different publication categories and were grouped as follows: articles (800—WoS, 691—Scopus); books (20—WoS, 37—Scopus); articles, proceeding papers (1—WoS); reviews (19—WoS, 16—Scopus); proceedings papers (557—WoS); and editorial materials (14—WoS, 11—Scopus). The analysed period was 2020–2021, capturing the whole pandemic period for both databases.

The WoS database authors with the Bucharest University of Economic Studies affiliation used 43,688 references, which were published in 254 sources and used 4367 different keywords. On the other hand, the Scopus database authors with same affiliation used 39,051 references, which were published in 241 sources, and used 2838 different keywords. Table 2 pointed out the existence of a strong collaboration between authors; 1935 authors shared the documents published in the WoS database, while 2800 authors shared the documents published in the Scopus database.

## 4. Empirical Results

### 4.1. Most Productive Sources

**Figure 3:**
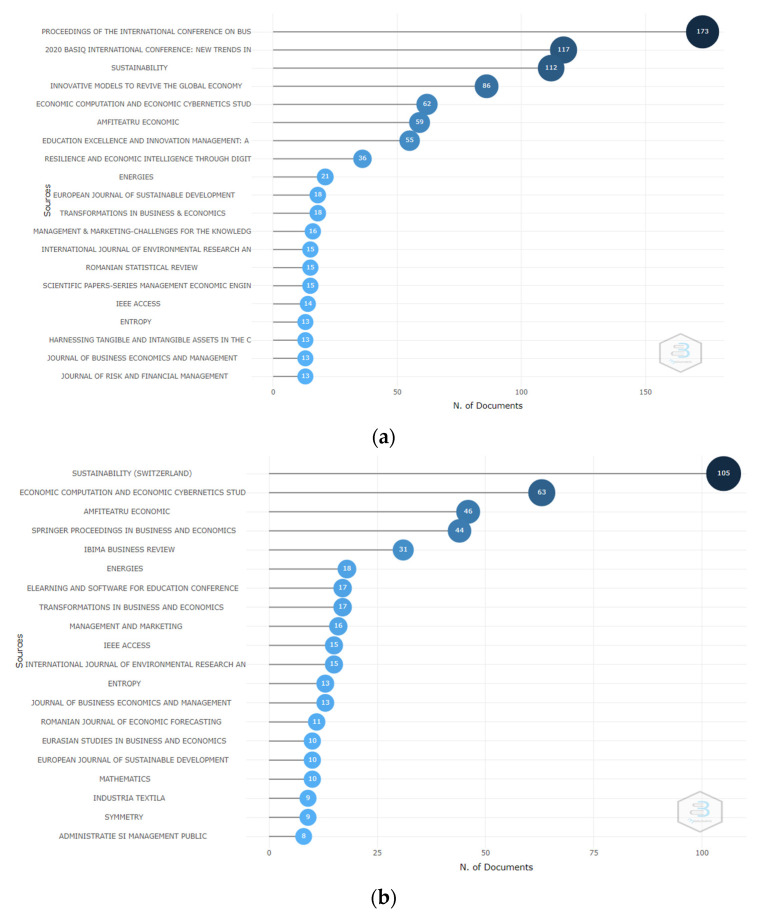
Twenty most productive sources. (**a**) WoS. (**b**) Scopus.

The most relevant journals that publish articles at the frontier of all topics have been analysed. Figure 3 explored the ranking of the twenty most abundant sources in the core collection of the Web of Science (WoS) and Scopus databases, highlighting that the terms used in the data collection design in the first place are the-Proceedings of the International Conference on Business Excellence and Economic Computation, Basiq International Conference (New Trends in Sustainable Business and Consumption), Sustainability, Economic Computation and Economic Cybernetics Studies, and Amfiteatru Economic for both platforms.

**Figure 4:**
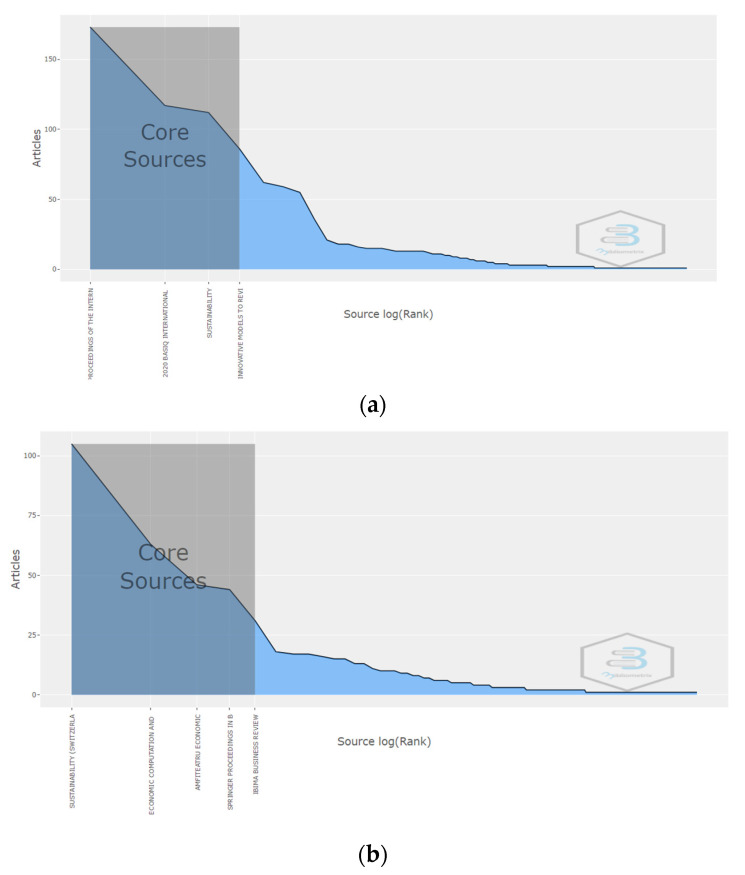
Bradford’s Law. (**a**) WoS. (**b**) Scopus.

According to Bradford’s Law, the first four most productive sources are the core sources out of the 254 records and represent the nucleus of journals published by the authors affiliated with the Bucharest University of Economic Studies. In Figure 4a, the core zone represents 37.4% of the total sources of the WoS database. It includes the following sources: Basiq International Conference: New Trends in Sustainable Business and Consumption 2020, Sustainability, Proceedings of the International Conference on Business Excellence and Economic Computation and Economic Cybernetics studies. In Figure 4b, the core zone represents 36.2% of the total sources of Scopus platform. It includes the following sources: Sustainability, Economic Computation and Economic Cybernetics Studies, Amfiteatru Economic, Springer Proceedings in Business and Economics and IBIMA Business Review.

Table 3 shows the list of most essential sources arranged in the decreasing order of h-index and with a value of h-index > 2. Sustainability and Lancet are the journals with the highest h-index of 12 and 9 on WoS and 11 and 10 on Scopus. Egghe pointed out the relevance of the g-index seen as an extension of the h-index and represents an essential tool for assessing the global citation performance [51]. Costas and Bordons pointed out that the g-index offers a better ranking position than the h-index [52]. Sustainability gains visibility and ranks first with the total number of citations (919 on WoS and 559 on Scopus), according to the total number of citations.

### 4.2. Most Cited Documents

Table 4 shows the list of most essential papers arranged in decreasing order by total citations. The most cited paper is Asymmetric dependence between stock market returns and news during COVID-19 financial turmoil published in 2020 in Financial Research Letters on WoS database, while the prevalence and attributable health burden of chronic respiratory diseases, 1990–2017: a systematic analysis for the Global Burden of Disease Study 2017 by Soriano et al., published in Lancet is the most cited paper on the Scopus platform.

### 4.3. Author Impact

**Figure 5:**
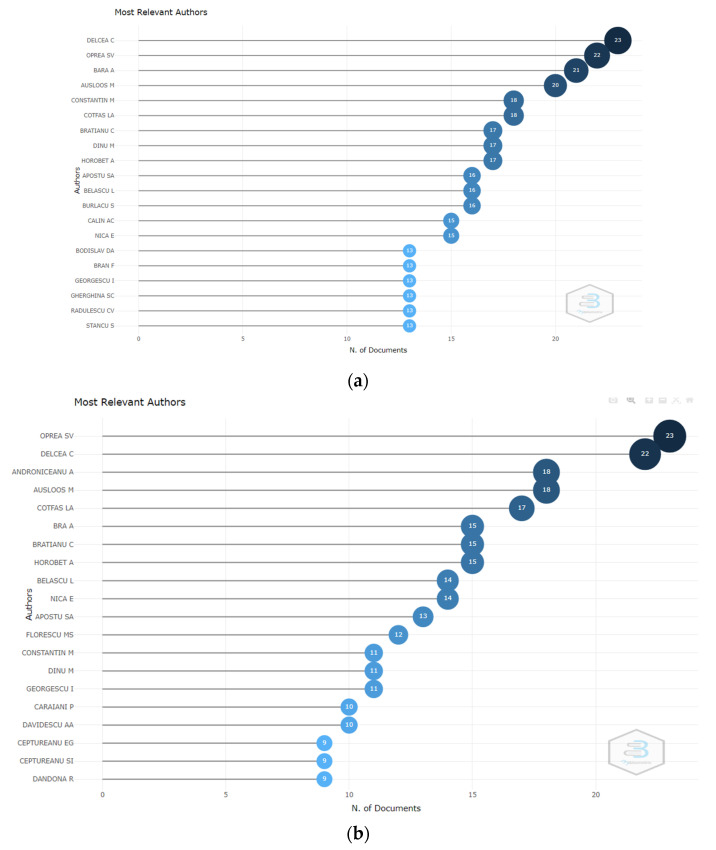
Most relevant authors by the number of documents. (**a**) WoS. (**b**) Scopus.

Among the most relevant authors by the total number of written documents that are affiliated with the Bucharest University of Economic Studies are Delcea C., Oprea S.V., Bara A., Herteliu C., Dinu M., and Andrei T. In terms of H-index, G-index, and M-index, Herteliu C., Andrei T., Pana A., Stefan S.C., and Mirica A. are the most influential authors. Similar orders present both outputs of the two databases used (Figure 5).

### 4.4. Three Plots Field and Word Growth

**Figure 6:**
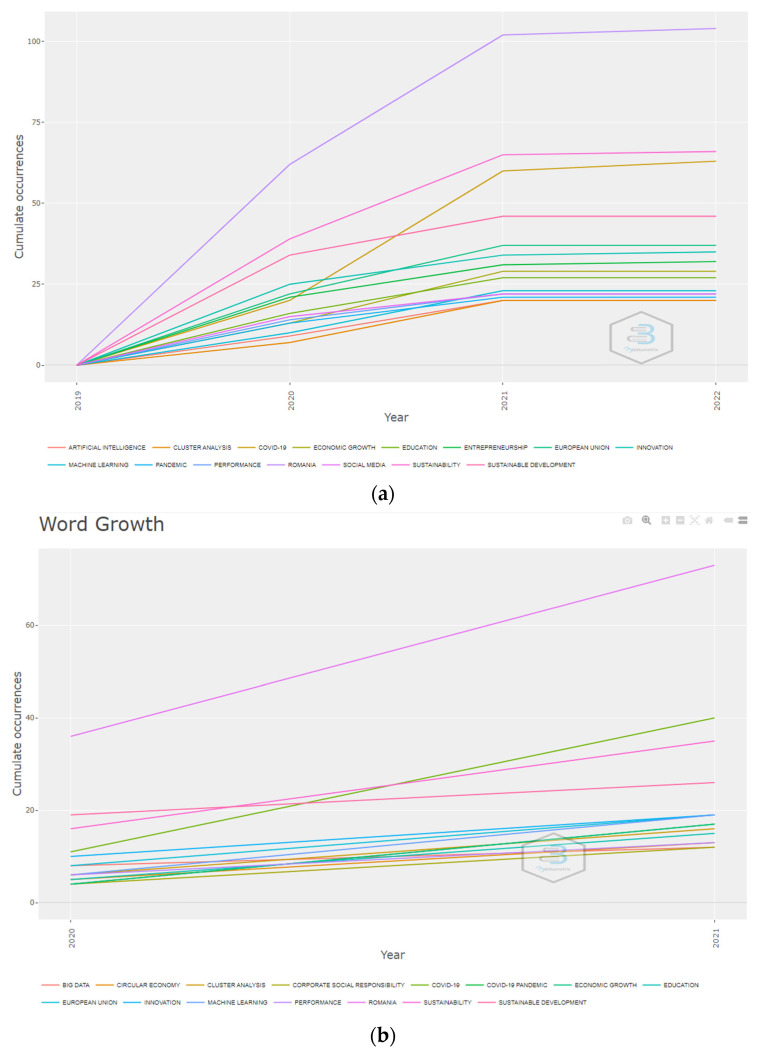
Most frequent keywords in the abstracts of publications. (**a**) WoS. (**b**) Scopus.

The evolution of the primary term occurrences revealed that the terms with the highest increase in occurrences have been “Romania”, “sustainability”, “COVID-19”, or “sustainable development” (Figure 6) for both the WoS and Scopus platforms.

**Figure 7:**
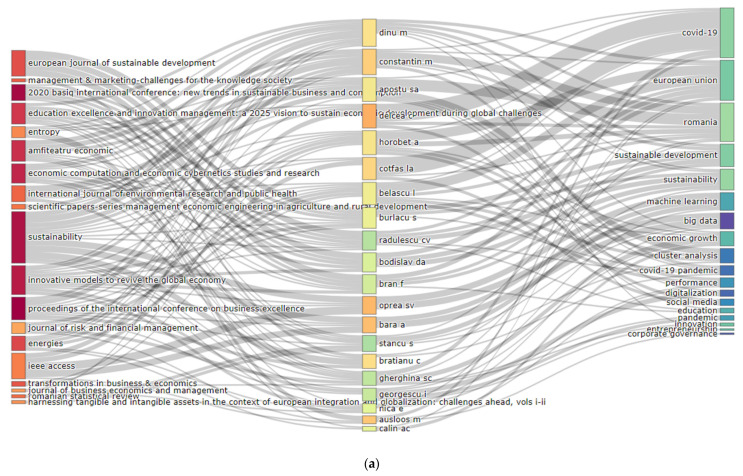

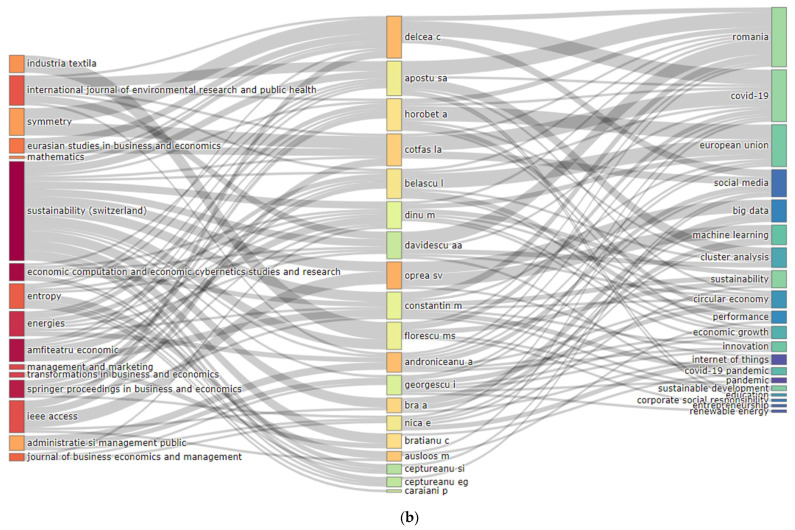
Three field plots. (**a**) WoS. (**b**) Scopus.

Valuable information has been provided by the Sankey diagram (Figure 7), analysing the relationship between sources, keywords, and authors, and the height of the rectangles offers information about the relations appearing between elements. The analysis addresses the research topics (keywords of the authors) of the Bucharest University of Economic Studies that have explored and the sources they have most often published in. Thus, on the WoS platform were found seven authors that published papers with one of the keywords, “sustainability” (Delcea C, Bratianu C, Dinu M, Florescu M, Constantin M, Ausloos M, and Burlacu S) in four journals (*IEEE Access*, *Sustainability*, *Economic Computation*, and *Economic Cybernetics Studies and Research, Energies*). In the context of the pandemic, the COVID-19 keywords were found in papers published by Delcea C, Cotfas L, Gherghina S.C., and Florescu M.S. in journals such as *IEEE Access*, *Economic Computation*, and *Economic Cybernetics Studies and Research*, *Amfiteatru Economic*, as well as in Education Excellence, and Innovation Management: a 2025 vision to sustain economic development during global challenges. Regarding the Scopus platform, it is pointed out that all the authors have published at least a paper in Sustainability with keywords such as “Romania”, “COVID-19”, “sustainability” or “circular economy”.

### 4.5. Cluster Analysi

#### 4.5.1. Abstracts’ World Cloud Analysis

**Figure 8:**
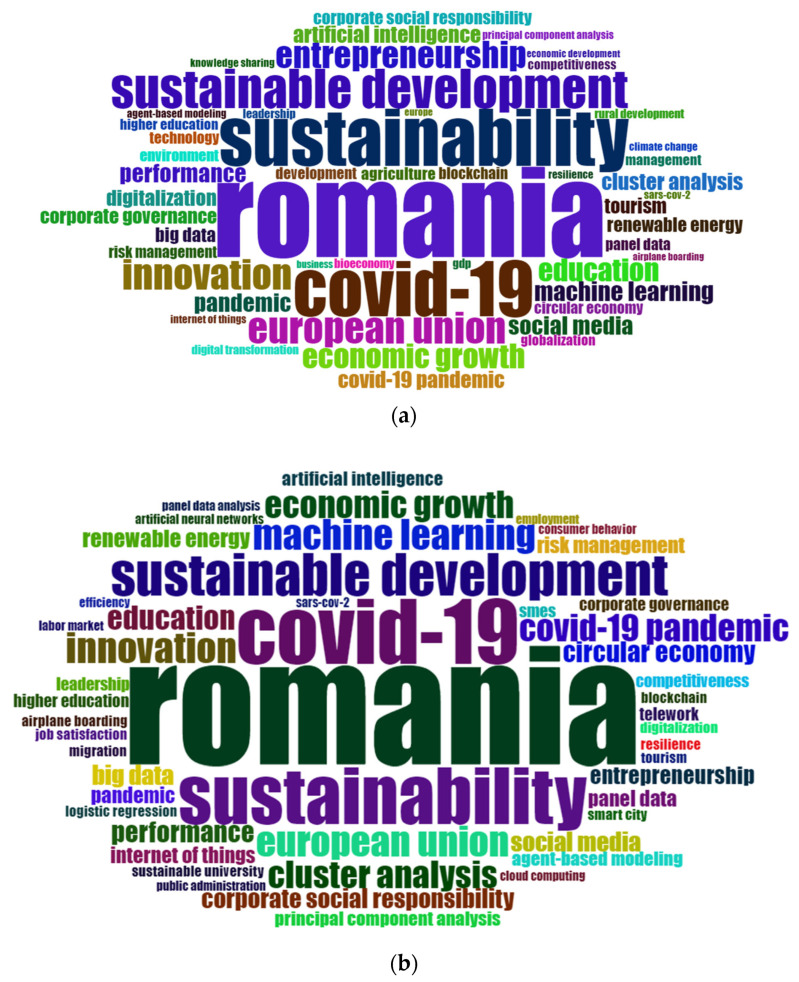
Most frequent keywords in abstracts of publications. (**a**) WoS. (**b**) Scopus.

Abstracts’ world cloud analysis explores the valuable information from publications abstracts, providing insight into the main topics and research trends. Figure 8a displays the top 50 of the most frequent keywords from the WoS database, revealing that the most frequent combinations are “Romania”, “sustainability”, “European Union”, “COVID-19”, “innovation”, “sustainable development” and “economic growth. Figure 8b displays the same top 50 but in this case from the Scopus database and reveals that the most frequent combinations of keywords are “Romania”, “sustainability”, “sustainable development”, “innovation”, “machine learning”, “COVID-19” and “education”.

#### 4.5.2. Authors and Documents Clustering by Coupling

**Figure 9:**
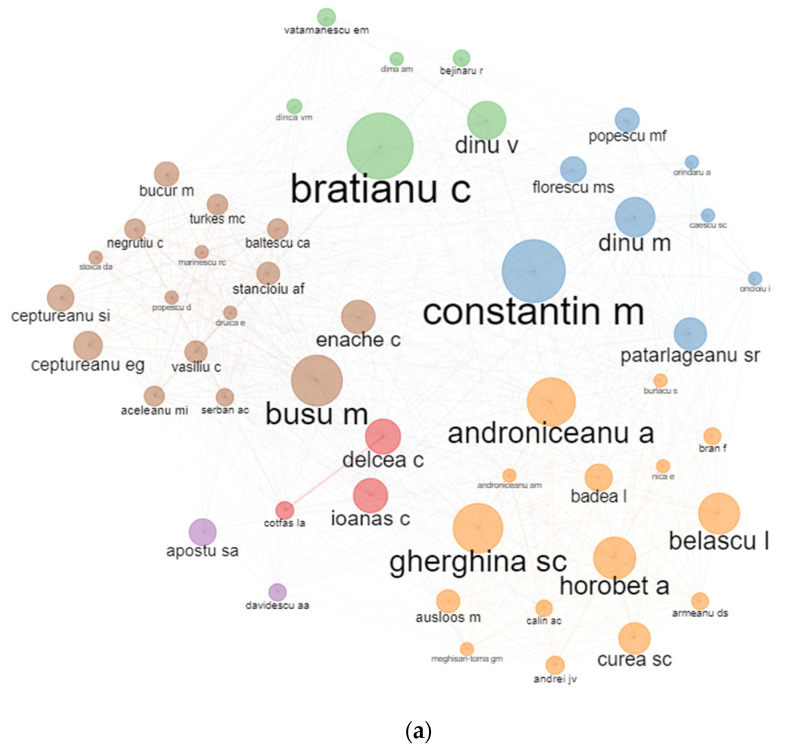

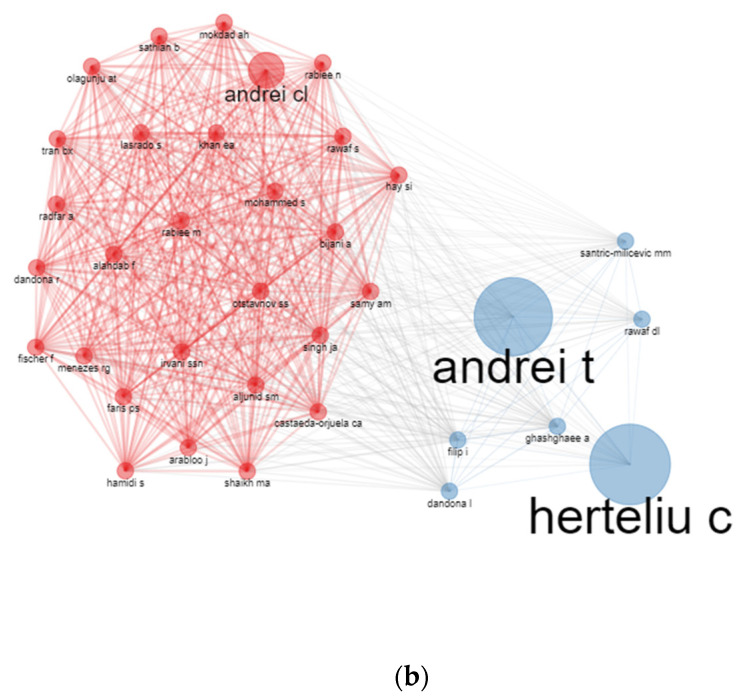
Authors’ clustering by coupling. (**a**) WoS. (**b**) Scopus.

The results of bibliographic coupling outline the current research perspectives in terms of the authors’ affiliation with the Bucharest University of Economic Studies (Figure 9). We apply no restrictions concerning the number of citations to grasp the entire dataset. The corresponding information enables an analysis of the authors publishing the relevant literature in the range of literature on the pandemic period 2020–2021. Six clusters of authors’ coupling were identified for the WoS database. The blue one consists of authors such as Constantin M., Popescu M.F., Dinu M., and Florescu M.S. and is led by terms like “circular economy”, “economic modelling”and “informality”. The green cluster consists in Bratianu C., Dinu V., and Vatamanescu E.M. and is labelled as management, performance, and business. In the yellow cluster, papers are identified with words such as ‘growth’, ‘risk’, and ‘co2 emissions’ concerning authors such as Androniceanu A., Gherghina S.C., Horobet A., Belascu L., and Armeanu D.S. The red cluster consisting of authors such as Delcea C., Ionas C., and Cotfas L.A. identifies words such as “optimization”, “simulation”, and “passengers”. Brown cluster is led by authors such as Busu M., Enache C, and Ceptureanu E.G. and has the main topics such as sustainability, sustainable development, or innovation. Purple cluster has the two authors Davidescu A.A. and Apostu S.A. and it is defined by keywords such as informal economy, digitalisation, and informality.

In terms of the Scopus database, only two main clusters were identified. The blue cluster has the main authors Herteliu C. and Andrei T. being defined by health and religion, while the red cluster has the central node the author Andrei C.L. and it is defined as health, medicine, COVID-19.

**Figure 10:**
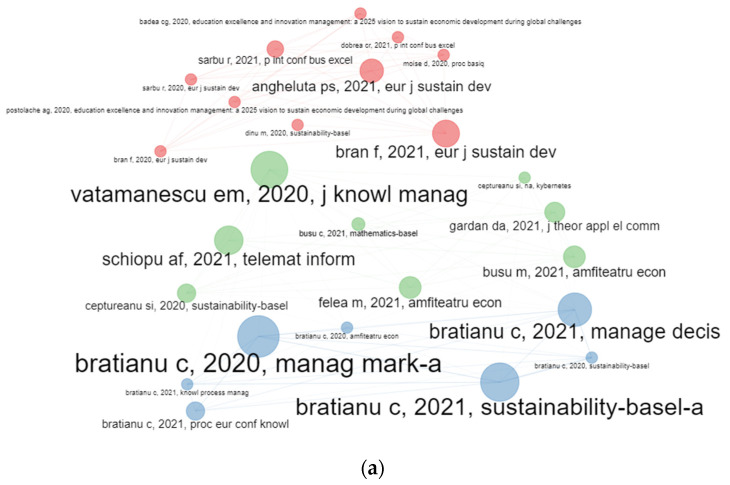

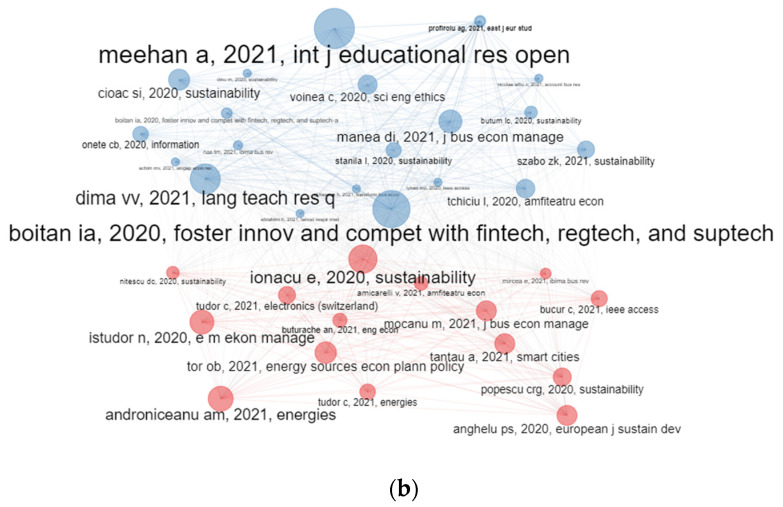
Papers’ clustering by coupling. (**a**) WoS. (**b**) Scopus.

The coupling map by papers reveals three clusters: the green one has as leading topics management, behaviour, and intention, the blue one focuses on papers treating heterogeneity, heuristics, and judgment, while the red one focuses mainly on cointegration, panel date, causality, and communities (Figure 10a). In Figure 10b are pointed out two clusters, the blue one focusing on education threats in times of pandemics, while the red one focuses on sustainable development goals.

### 4.6. Conceptual Structure

The conceptual structure includes network analysis relying on keyword co-occurrence and multiple correspondence analysis illustrating the major research themes.

#### 4.6.1. The Conceptual Structure Map of Major Themes Using MCA

The conceptual structure map of the Bucharest University of Economic Studies researchers primary topics, separating the most common terms based on mapping the link between one word and another through area mapping, has also offered useful information. To create a mapping between words with similar values, each word is inserted according to the values of Dim 1 and Dim 2. Negatively correlated variable categories are positioned in the opposite quadrants of the plot origin, whereas positively correlated variable categories are clustered together in MCA. The variable quality is determined by the distance between category points and the origin on the factor map.

**Figure 11:**
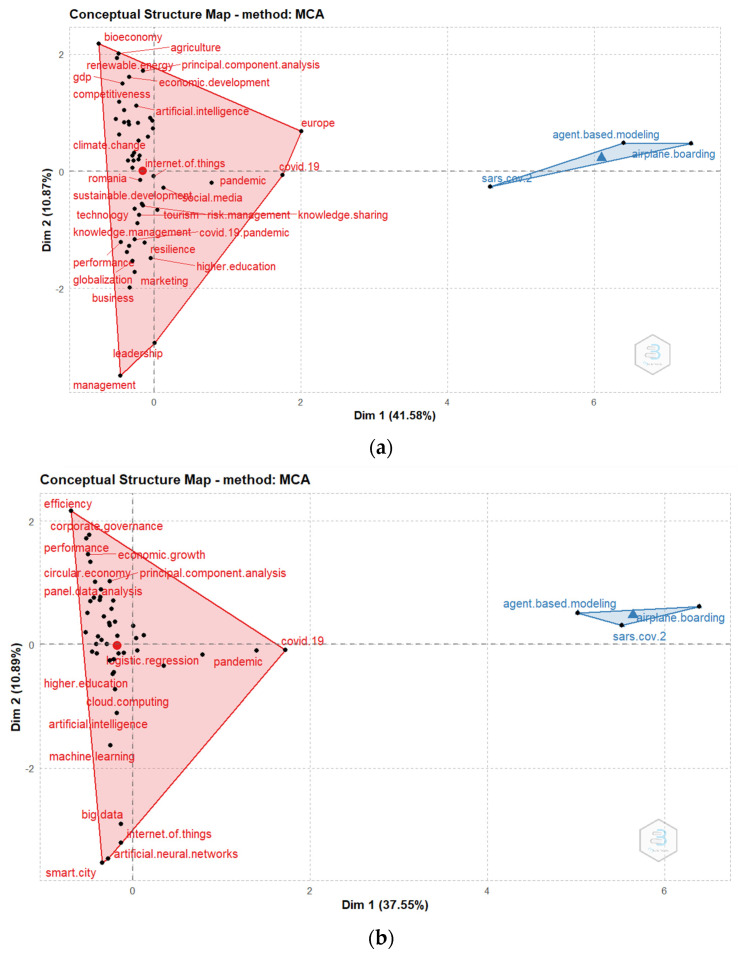
Conceptual structure map of Bucharest University of Economic Studies researchers. (**a**) WoS. (**b**) Scopus.

We can distinguish between two main clusters for both platforms with similar results: the bigger red cluster consisting of various elements of economic development, sustainability, and pandemic, including GDP, economic development, economic growth, sustainability, COVID-19, etc., and the second blue cluster more related to airplane boarding simulations, including SARS-CoV-2, agent-based modelling and airplane boarding (Figure 11).

#### 4.6.2. Analysis of Keyword Co-Occurrence

**Figure 12:**
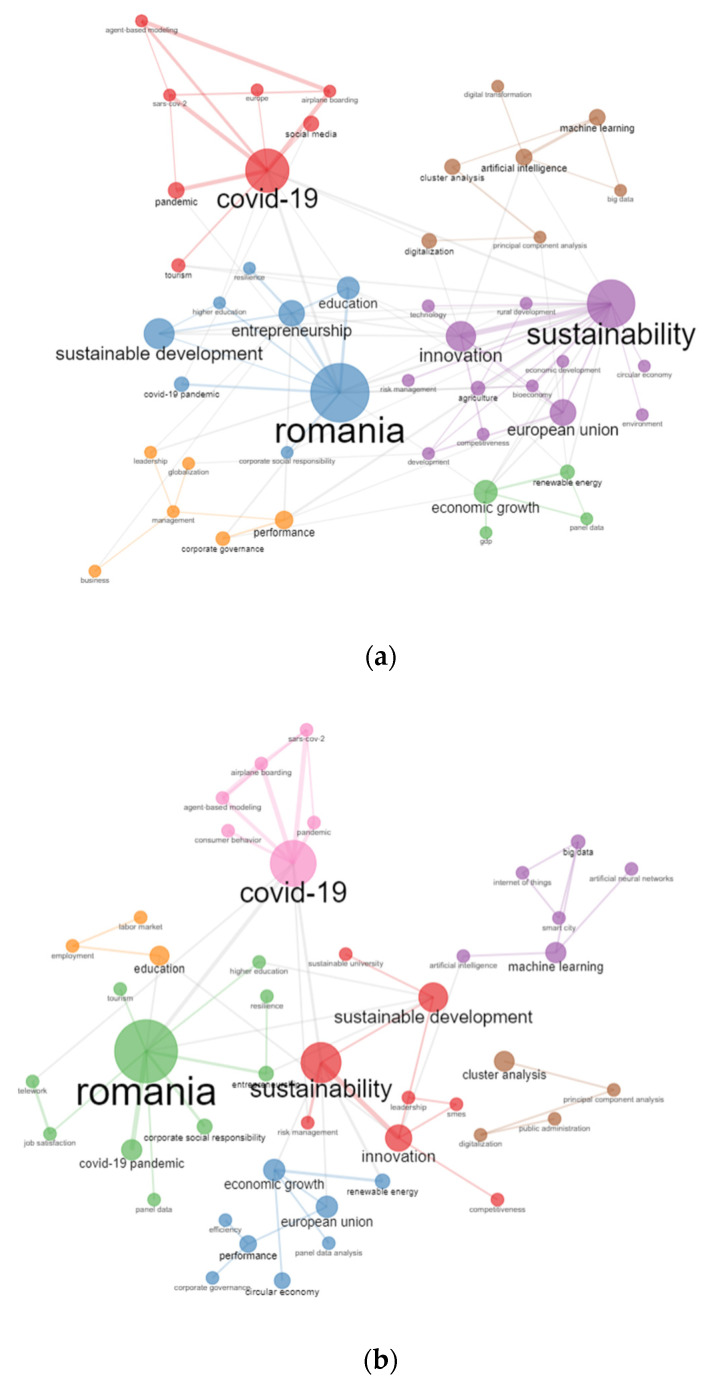
Cooccurrence network analysis on authors’ keywords. (**a**) WoS. (**b**) Scopus.

The keyword cooccurrence network displayed in Figure 12 highlights the number of occurrences of the keywords through the dimension of the box, while topic similarity is represented by the distance between the elements of individual pairs. In the analysis, six different box colours have been highlighted signalling six individual clusters, the focal points of the network being Romania, sustainability, COVID-19, European Union, performance, and machine learning in the case of the WoS database. The Scopus database pointed out seven different box colours having as the main focal points of the network the following keywords: COVID-19, Romania, sustainability, machine learning, cluster analysis, European Union, and education.

### 4.7. Intellectual Structure Based on Co-Citation Analysis

**Figure 13:**
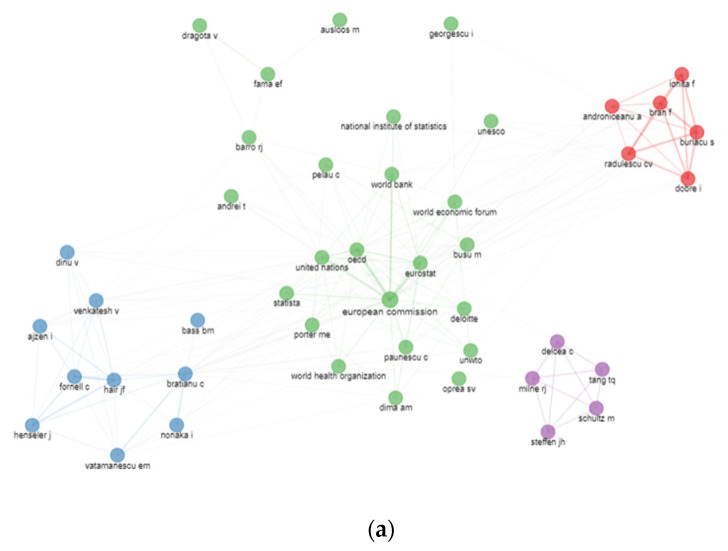

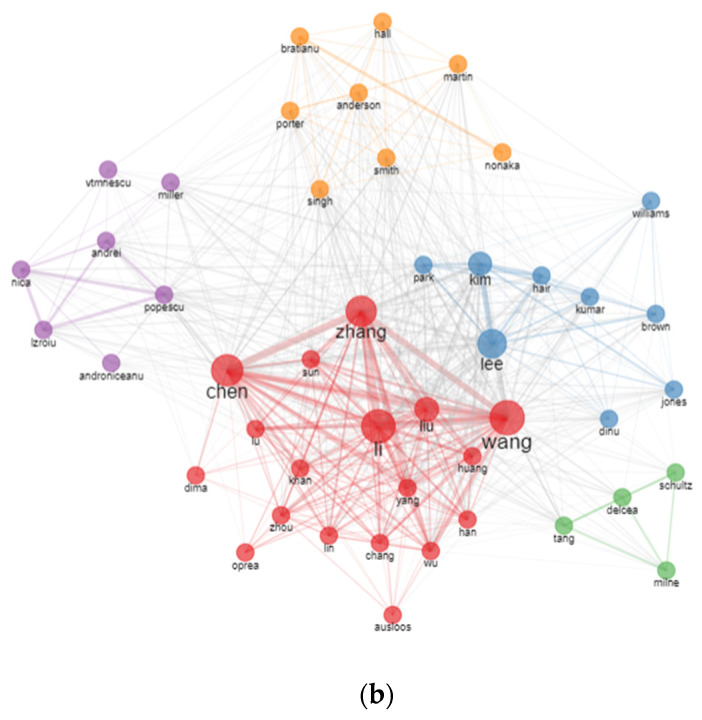
Co-citation network analysis on authors. (**a**) WoS. (**b**) Scopus.

Figure 11, Figure 12 and Figure 13 exhibit the co-citation analysis, with each box representing an author/article or journal with papers with at least one author affiliated with the Bucharest University of Economic Studies, the size of the box revealing the volume of the citation (the larger the box, the more author’s documents are cited), while the proximity of the boxes indicates a close relationship between the co-cited documents.

#### 4.7.1. Co-Citation Analysis on Authors

The co-citation analysis among authors for the body of literature investigating at least one author affiliated with the Bucharest University of Economic Studies reveals four main clusters with four main authors from the WoS database, European Commission, Burlacu S., Bratianu C., Ausloos M., and Delcea C. and their linkages with the group of authors from their subnetwork. European Commission, Androniceanu A., Bratianu C., and Delcea C. are the nodes with the highest betweenness with respect to their cluster, showing how important these authors are in term of the average pathway between other pairs of authors. In terms of closeness, centrality measure, and eigenvector centrality, the same five authors have shared the highest values.

By clusters, among the most relevant authors in the green cluster, we can mention European Commission, OECD, and Eurostat, in the red cluster Androniceanu A., Radulescu C.V, and Burlacu S., while, in the blue cluster, the reference names are Bratianu C., Vatamanescu E.M., and Dinu V. The purple cluster has the most relevant authors such as Delcea C. and Mine R.J.

In terms of the Scopus database, five main clusters were identified with five main authors such as Wang, Delcea, Androniceanu, Bratianu, and Lee. The red cluster has as reference names authors like Wang, Chen, Li, or Zhang, while the blue one has Lee, Kim, or Dinu as the nodes with the highest centrality value. The yellow cluster has the principal authors Bratianu, Porter, or Nonka; the purple one has Androniceanu, Popescu and Andrei the nodes with highest value of centrality; and the green cluster is similar with the purple cluster from the WoS database with authors such as Delcea, Mine, and Schultz as the central nodes (Figure 13).

#### 4.7.2. Co-Citation on Papers

The co-citation analysis among papers for the body of literature researching the Bucharest University of Economic Studies has identified three main clusters with primary papers, Dobre (2019), Delcea (2018), Fornell (1981), and Dragota (2014), and their linkages with the set of papers from their subnetwork. The nodes with the highest betweenness are Dobre (2019), Radulescu (2018), Ionita (2019), Delcea (2018), and Bratianu (2020), indicating how important these papers are in terms of the average pathway between other pairs of paper. The same five papers have had the greatest values for proximity, centrality measure, and eigenvector centrality.

By clusters, the most relevant papers in the blue cluster are Delcea (2018), Schultz (2018), and Delcea (2019); in the red cluster, Dobre (2019), Radulescu (2018), and Ionita (2019); and in the green cluster, Fornell (1981), Bratianu (2019), and Bratianu (2020) are the reference documents.

**Figure 14:**
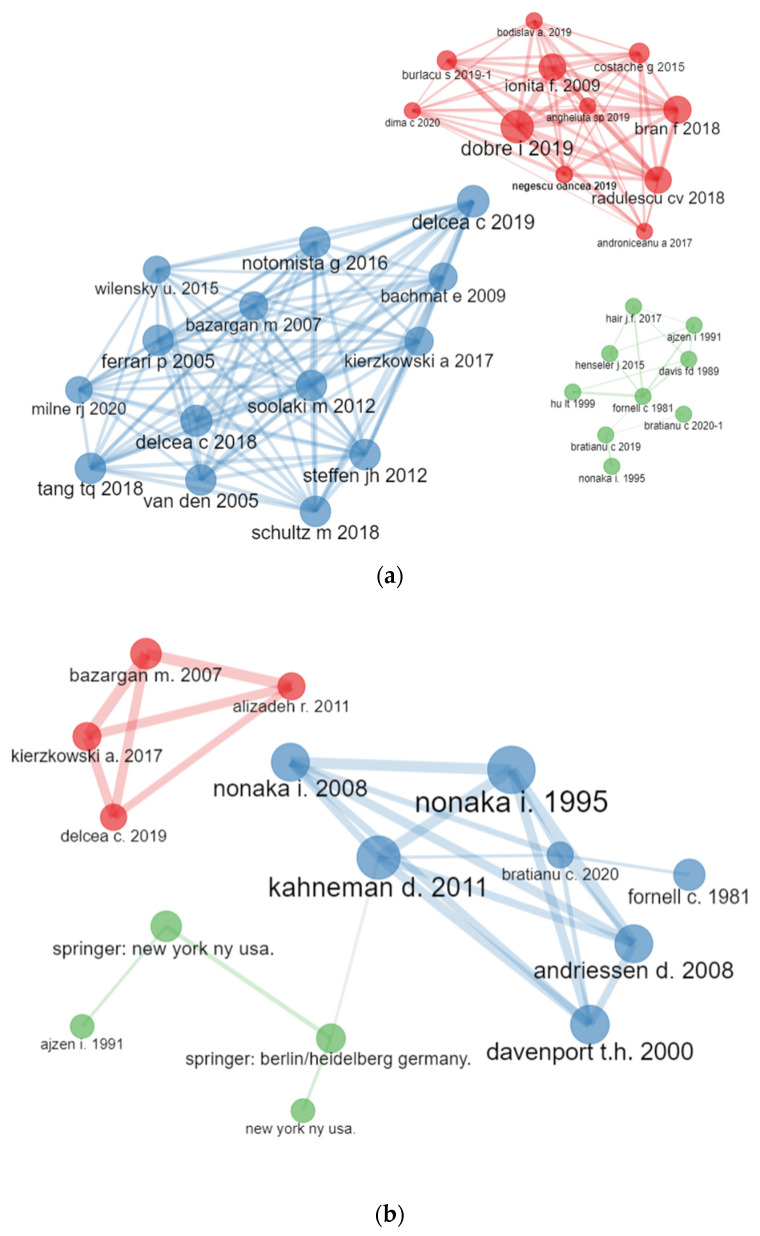
Co-citation network analysis on paper. (**a**) WoS. (**b**) Scopus.

Similarly, the Scopus database pointed out three main clusters with the main nodes from each one being Nonaka (1995) for the blue cluster, Delcea (2019) for the red cluster, and Ajzen (1991) in the green cluster Figure 14.

#### 4.7.3. Co-Citation on Journals

**Figure 15:**
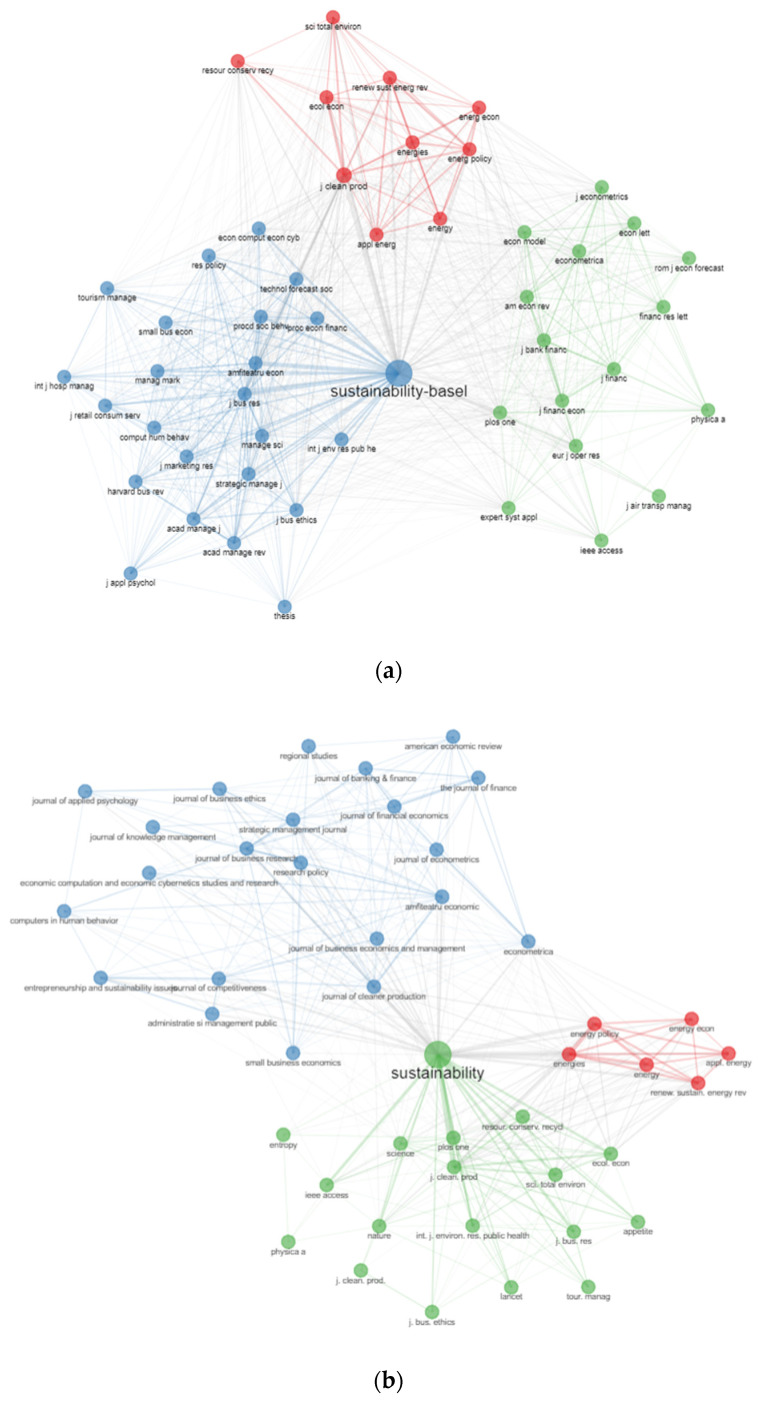
Cooccurrence network analysis in journals. (**a**) WoS. (**b**) Scopus.

The co-citation analysis of journals in the range of the literature on the Bucharest University of Economic Studies affiliated papers has identified three main clusters with primary journals: *Journal of Banking and Finance, American Economic Review, PLOS One* and *Economic Modelling* are in the blue cluster, while, in the red cluster, there are journals such as the *Journal of Cleaner Production, Renewable & Sustainable Energy Reviews, Energies, Ecological Economics and Resources*, and *Conservation & Recycling*, while, in the green cluster, *Sustainability, Journal of Business Research, Procedia Economics and Finance*, and *Amfiteatru Economic*. The above-mentioned journals indicate the importance in terms of the average pathway between other pairs of journals and show the greatest values from proximity (Figure 15). Similar results were provided also by the Scopus database.

### 4.8. Social Structure through Collaboration Network Analysis of Authors, Institutions, and Countries

**Figure 16:**
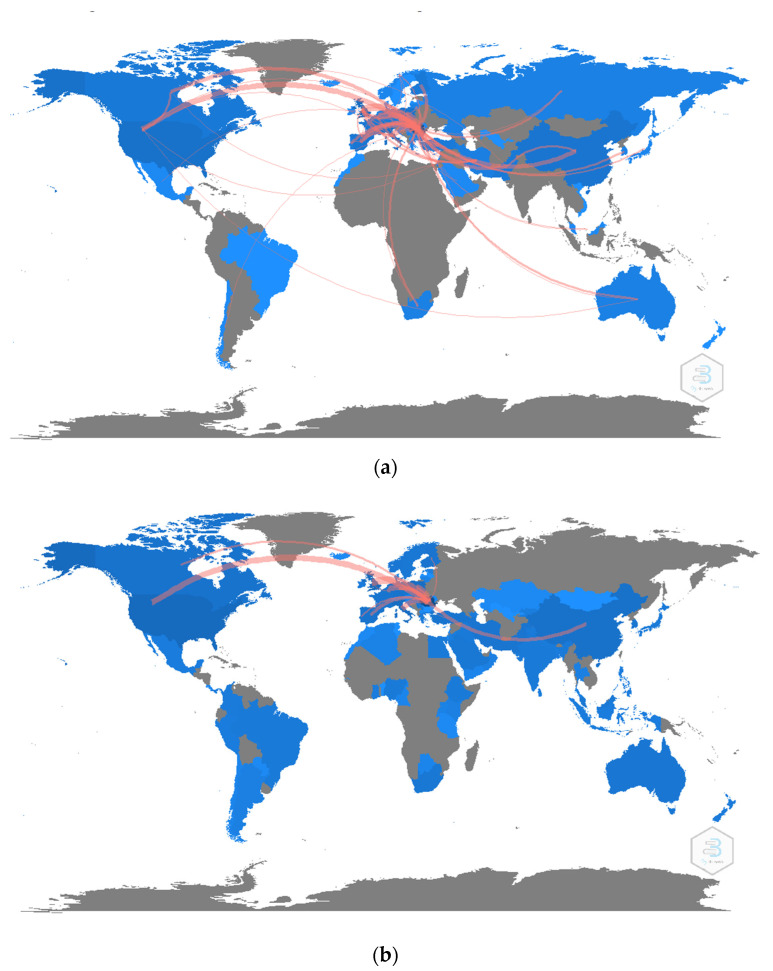
Collaborative network of countries. (**a**) WoS. (**b**) Scopus.

Figure 16 shows the country collaboration map, highlighting those relationships between US, UK, Canada, Spain, and Italy when we are referring to the Bucharest University of Economic Studies authors. Two countries hold a connection line indicating the status of collaboration among them. The scale of cooperation is represented through the thickness of the line [53]. Both platforms gave similar results in terms of country collaboration.

#### 4.8.1. Authors Collaboration

**Figure 17:**
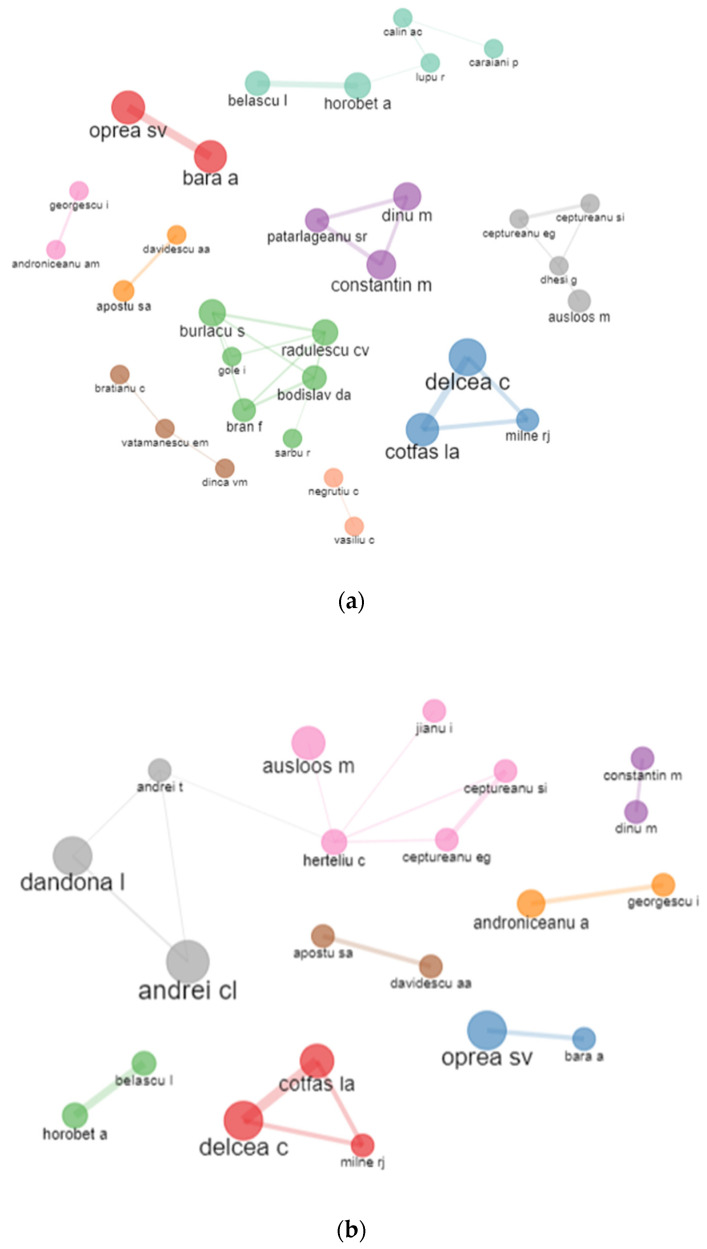
Authors collaboration network. (**a**) WoS. (**b**) Scopus.

Figure 17 reveals the main collaboration between the Bucharest University of Economic Studies authors. Several clusters of authors have been found in the analysis mentioning 11 clusters of sub-networks. Herteliu C., Andrei T., Pana A., Mirica A., and Stefan S.C. are part of the green cluster meaning that they usually are the authors on the same papers. Delcea C., Ionas C., and Cotfas L.A. are part of the same cluster of authors. Oprea S.V. and Bara A. usually collaborate on papers, as well as Davidescu A.A. and Apostu S.A. Similar results were produced by both platforms.

#### 4.8.2. Institution Collaboration

**Figure 18:**
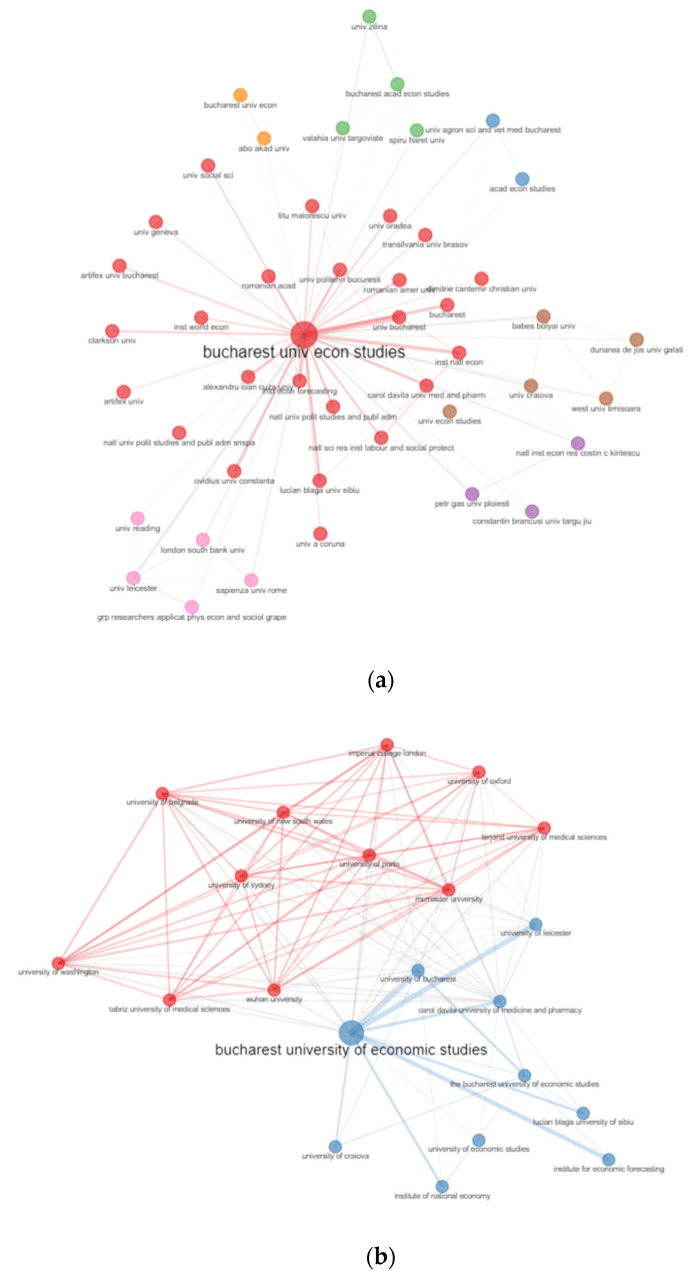
Institution’s collaboration network. (**a**) WoS. (**b**) Scopus.

Figure 18a reveals the main collaboration between institutions of the analysed papers. The strongest connection with the Bucharest University of Economic Studies are University of Bucharest, Carol Davila University of Medicine and Pharmacy, University of Žilina, University of Leicester, Politehnica University of Bucharest, and The Institute for Economic Forecasting. Figure 18b reveals the main collaboration between institutions were Bucharest University of Economic Studies with more international collaborations with universities such as Wuhan University, University of Sydney, University of Porto to points out some of the collaborations.

## 5. Discussion

SARS-CoV-2 syndrome has forever changed the scientific activity of teachers/researchers and, thus, the quality of life. The main aim of the research has been to explore the new research directions of the professors and researchers of the Bucharest Academy of Economic Studies in response to the health crisis using the bibliometric analysis of the publications in the field extracted from two relevant databases, WoS and Scopus.

The empirical results lead to the conclusion that the pandemic has created this boost in the number and quality of medical publications for the professors at the Bucharest University of Economic Studies who created now new multidisciplinary teams. Also, the analysis based on the publications extracted from both databases revealed that approximately 78% of WoS is covered in Scopus database and the analysis provided similar results.

Both platforms tackled the same productive sources, while according to the Bradford Law, the core zone is almost the same in both platforms. *Sustainability* is the journal which gains visibility and ranks first with a total number of citations (919 on WoS and 559 on Scopus) according to the total number of citations.

In terms of the most cited documents, it is worth to mention that in the WoS platform, the most cited paper was by excellence a Core Economics one tackling the asymmetric dependence between stock market returns and news during COVID-19 financial turmoil and it has been published in 2020 in Financial Research Letters. At the opposite side, in Scopus database, the most cited document is a medical paper treating the prevalence and attributable health burden of chronic respiratory diseases, 1990–2017: a systematic analysis for the Global Burden of Disease Study 2017, published in Lancet.

From the perspective of the author impact, both platforms WoS and Scopus revealed that same group of prolific authors: Herteliu C., Andrei T., Pana A., Stefan S.C., and Mirica A.

Based on the results of both platforms, during the pandemic, most was written on topics such as “COVID-19”, “innovation”, “sustainable development”, “economic growth”, “Romania”, “sustainability” and “Euro-pean Union”.

The conceptual structure analysis revealed the existence of two clusters for both platforms with similar results: the bigger red cluster consisting of various elements of economic development, sustainability, and pandemic, including GDP, economic development, economic growth, sustainability, COVID-19, etc., and the second blue cluster more related to airplane boarding simulations, including SARS-CoV-2, agent-based modelling, and airplane boarding.

During the pandemic, the Bucharest University of Economic Studies intensifies the institutional collaboration with different organizations, among which: The University of Bucharest, Carol Davila University of Medicine and Pharmacy, University of Žilina, University of Leicester, Politehnica University of Bucharest, and The Institute for Economic Forecasting, as well as with Wuhan University, University of Sydney, and University of Porto as international collaborations.

From the perspective of the research activity, there are several types of opportunities that the pandemic has brought at national and international level. A first category is related to the research process, where there is a better collaboration: increasing the solidarity of institutions and researchers from different parts of the country and the world, a greater desire to collaborate in the context in which humanity is fully facing a new, negative phenomenon that requires a coordinated response, faster communication using digital platforms, and awareness of the importance of interdisciplinary topics. Researchers worked in international teams generating added value increasing their work well-being, each specialist coming with his own experience from the country of origin and culture.

The second category is related to the need for research, which leads to an increase in the intensity of activity in this sector: the need for studies on the impact of the pandemic on the individual level, organizational level, and human society. Most research during this period has focused on the impact of the pandemic and tackled more on multidisciplinary research (economics, medicine, and pharmacology).

Investing the number of articles published during the pandemic period in both databases using the affiliation of Bucharest University of Economic Studies, it can be highlighted that, the pandemic caused the number of multidisciplinary articles to increase significantly (by 73% in the period 2020–2021, compared to 2018–2019). The joining of at least two main fields of research during this period, economics, and medicine, as well as the collaboration of researchers from the two areas of study, led to a better understanding of the pandemic and its effects on economics and medicine. The exchange of information and knowledge between researchers from different fields of research, apparently unrelated, have positively influenced the quality of the articles. Finally, we can talk about a transfer of knowledge from one field of research to another, which can lead to an improvement of research skills and techniques. All this positively influencing the researchers’ publishing activity. For teachers/researchers, an important side of publishing activity resides on the quality of published papers and on the scientific performance of the accessed journals and this is a consequence of the pandemic even at the level of multidisciplinary publications who significantly developed during this period, bringing an added value to the works published by the researchers from BUES.

The COVID-19 pandemic has led to an improvement in the professional for the academic personnel of Bucharest University of Economic Studies for several reasons:

i. The need for information, which leads to increased interest in research.
ii. Increasing interest in certain interdisciplinary research fields (economics and medicine);
iii. Wider national and international collaboration.

The world has changed, and people in general tend to become deeper, more analytical when their universe is shaken by a pandemic or a war. Practically in these critical moments we can witness new scientific discoveries, the most creative solutions to the problems that society faces and an improvement in the end of the professional performance and quality of life of teachers. In this context, scientific research plays an important role.

No doubt we can ask questions about what the world will look like when we don’t return to normal and whether the SARS-CoV-2 topic will remain a favourite topic of research.

## 6. Conclusions

The SARS-CoV-2 syndrome has had a significant impact upon research in all activity fields. The Bucharest University of Economic Studies (BUES) is one of the most prestigious universities in Romania. This pandemic has led to a large part of the research professors at USEB focusing all their research activity on this topic; with the approach focused more on documenting, relying on secondary sources, rather than on direct research.

The main aim of the research has been to explore the new research directions of the professors and researchers of the Bucharest Academy of Economic Studies in response to the health crisis using the bibliometric analysis of the publications in the field extracted from two relevant databases WoS and Scopus for the period January 2020–December 2021.

Most of the research topics of BUES professors and researchers over the past two years have been related to the crisis caused by SARS-CoV-2 syndrome and how it has affected the quality of life. Through the research topics approached, an attempt was made to measure the economic and social impact generated by this pandemic.

The empirical results lead to the conclusion that the pandemic has created this boost in the number and quality of medical publications for the professors at the Bucharest University of Economic Studies who created now new multidisciplinary teams. Also, the analysis based on the publications extracted from both databases revealed that approximately 78% of WoS is covered in Scopus database and the analysis provided similar results.

Bibliometric analysis helps us better understand the scientific activity of the BUES researchers during 2020–2021. The professors published more locally, either in conferences held within BUES or in BUES journals and opted more for multidisciplinary journals. Also, during this period, there was an increased predilection towards citations from medical journals (e.g., Lancet), due to the topic being addressed. Among the more frequently used key words in publication abstracts, we highlight: “Romania”, “sustainability”, “European Union”, “COVID-19”, “innovation”, and “economic growth”, “machine learning” or “entrepreneurship”. The terms that saw the highest rate of increase in the articles published by USEB researchers are: “COVID-19”, “Romania”, “sustainable development”, or “sustainability”, due to the journals in which they publish regularly (IEEE Access, Sustainability, Economic Computation and Economic Cybernetics Studies and Research, Energies).

The analysis of the major research topics from January 2020 to December 2021 revealed two clusters: economic development, sustainability, and pandemic, GDP, economic growth, sustainability, management, education, occupation, competitiveness, convergence, COVID-19 (the first cluster) and SARS-CoV-2, agent-based modelling, and algorithms for optimizing passenger boarding (the second cluster).

Basically, the pandemic period seems to not have affected too strongly the intensity of the research activities, but there are aspects where a change has been felt, such as, for example, the way of interacting with the different factors interested in research. There are no major changes in the direction of the research either, but rather adaptations by considering the influence of the new context on the research activity and domains, as well as more nuances in the topics researched, from the perspective of the influences generated by the impact of the pandemic.

From the perspective of research activity, several types of opportunities arise that the pandemic has brought to the national and international level. A first category refers to the research process, where there is better collaboration between researchers from different parts of the country and from around the world, a conclusion also validated by Lee, Haupt [13], Raman [14]. The second category refers to the need for research, which leads to the intensification of activity in this sector: the need for studies on the impact of the pandemic on the individual, organizations, and human society. There is also a third category of opportunities; however, it is treated rather critically, and we are referring here to the forced digitalization of recent, which has favoured online studies. Last, but not least, during the pandemic, the number of multidisciplinary articles published by the researchers of Bucharest University of Economic Studies increased significantly.

The value of this work resides in the way it captures the concerns of Romanian researchers during the pandemic, concerns that converge towards the trend that manifests itself at international level during this period.

In terms of the main limitations and future directions of research, it can be mentioned that it does not capture the social and psychological impact that the researchers have felt on a personal level, as well as because of the difficulties in collaboration (considering the restriction of direct interactions and the migration to the online environment). The limits also refer to the topics addressed, which remained focused mainly on the impact of the pandemic, analysing articles published in the WoS and Scopus. Therefore, future studies need to be expanded to the following academic platforms Google Scholar, EBSCO, and PubMed, as well as to publications of Romanian researchers from other universities/research institutions.

## Figures and Tables

**Table 1:** An overview of the most relevant studies of research publications in a university-based approach.

Authors	Method	Results
Martinez-Lopez, Merigo, Valenzuela-Fernandez, Nicolas (2018) [19]	Bibliometric analysis on Scopus database of publications between 1967–2017 of European Journal of Marketing	British writers and institutions are the most productive in the European Journal of Marketing in the number of articles published. In the medium term, nations such as France, Germany, Italy, and Spain, should perform similarly to those of the United Kingdom.
Maharana, Bihari (2013) [20]	Bibliometric evaluation of Sambalpur University’s scientific research output from 2007 to 2011.	Chemistry is the most popular research field, followed by Physics, Astronomy, Plant Science, Engineering.
Podsakoff, MacKenzie, Podsakoff, Bachrach (2008) [21]	Bibliometric analysis of 30 journals of management between 1981–2004.	The most significant influences on university publications were university size, the number of PhDs given, research expenditures, and endowment assets.
Soytas (2021) [22]	Bibliometric analysis	“COVID-19” and “older people” were the most often used terms, and they were significantly associated with “social isolation”, “dementia”, “mortality”, and “loneliness”.
Gaviria-Marian, Merigo, Popa (2018) [23]	The technique consists of performance analysis and a Journal of Knowledge Management (JKM).	The United States and the United Kingdom are the most prolific authors in this magazine. Emerging economies have a low participation rate in JKM.
Siwach, Seema (2018) [24]	The study aims to find out the publication trends in CCS Haryana Agricultural University (CCSHAU) university during 2001–2015.	Between 2001 and 2015, 2649 papers were published with 15,282 citations. Almost 47 percent of the university’s research was published in 10 journals.
Belter, Garcia, Livinski, Leon-Velarde, Weymouth, Glass (2019) [25]	Web of Science papers with at least one author linked with a Peruvian institution between 1997–2016	Between 1997 and 2016: the annual number of publications from Peru grew nine-fold; 66% of the publications included co-authors from the United States, 13% from the United Kingdom, 11% from Brazil, and 10% percent from Spain. 33% of the papers cited Universidad Peruana Cayetano Heredia (UPCH) as the primary research institution.
Zhou, Chen (2020) [26]	Bibliometric analysis on The Web of Science core collection database between 1 January 2000 and 17 March 2020	Nine thousand forty-three coronavirus publications were published in 1202 journals from 123 countries between 1 January 2000, and 17 March 2020. The United States provided the most articles, followed by China.

**Table 2:** Main information of the documents. (**a**) WoS. (**b**) Scopus.

**(a)**	
**Description**	**Results**
Sources (Journals, Books, etc)	254
Documents	1411
Average years from publication	1.56
Average citations per documents	2.12
Average citations per year per doc	0.83
References	43,688
Article	800
Book	20
Editorial material	14
Article, proceedings paper	1
Proceedings paper	557
Review	19
Keywords Plus (ID)	1557
Author’s Keywords (DE)	4367
Authors	2141
Author Appearances	4326
Authors of single-authored documents	206
Authors of multi-authored documents	1935
Documents per Author	0.64
Authors per Document	1.55
Co-Authors per Documents	3.13
Collaboration Index	1.75
**(b)**	
**Description**	**Results**
Sources (Journals, Books, etc)	241
Documents	876
Average years from publication	1.45
Average citations per documents	4.14
Average citations per year per doc	1.59
References	39,051
Article	691
Book	37
Editorial material	11
Article, proceedings paper	121
Review	16
Keywords Plus (ID)	2039
Author’s Keywords (DE)	2838
Authors	2889
Author Appearances	5608
Authors of single-authored documents	89
Authors of multi-authored documents	2800
Documents per Author	113
Authors per Document	3.41
Co-Authors per Documents	6.62
Collaboration Index	3.81

**Table 3:** Most important sources. (**a**) WoS. (**b**) Scopus.

Source	H-Index	G-Index	M-Index	TC	NP	PY_Start
**(a)**						
*SUSTAINABILITY*	12	19	4	919	93	2020
*LANCET*	9	13	4.5	648	72	2020
*IEEE ACCESS*	7	10	2.33	162	40	2020
*AMFITEATRU ECONOMIC*	6	9	2	162	40	2020
*ENERGIES*	5	8	1.66	77	16	2020
*INTERNATIONAL JOURNAL OF ENVIRONMENTAL RESEARCH AND PUBLIC HEALTH*	5	10	1.66	105	12	2020
*MANAGEMENT & MARKETING-CHALLENGES FOR THE KNOWLEDGE SOCIETY*	5	9	1.66	103	14	2020
*ECONOMIC COMPUTATION AND ECONOMIC CYBERNETICS STUDIES AND RESEARCH*	4	5	1.33	65	29	2020
*JOURNAL OF BUSINESS ECONOMICS AND MANAGEMENT*	4	6	1.33	47	6	2020
*KYBERNETES*	4	6	1.13	76	9	2021
(**b**)						
*SUSTAINABILITY*	11	17	3.66	559	84	2020
*LANCET*	10	12	3.33	472	73	2020
*IEEE ACCESS*	8	11	2.66	142	14	2020
*AMFITEATRU ECONOMIC*	5	9	1.66	132	31	2020
*ENERGIES*	5	7	1.66	69	14	2020
*MANAGEMENT AND MARKETING*	5	8	1.66	73	14	2020
*ECONOMIC COMPUTATION AND ECONOMIC CYBERNETICS STUDIES AND RESEARCH*	4	5	1.33	69	26	2020
*INTERNATIONAL JOURNAL OF ENVIRONMENTAL RESEARCH AND PUBLIC HEALTH*	4	10	1.3	120	10	2020
*JOURNAL OF BUSINESS ECONOMICS AND MANAGEMENT*	4	7	1.33	56	9	2020
*ADMINISTRATIE SI MANAGEMENT PUBLIC*	3	7	1	72	7	2020

**Table 4:** Most important papers. (**a**) WoS. (**b**) Scopus.

**(a)**	
**Paper**	**Total Citations**
CEPOI CO, 2020, FINANC RES LETT	88
FONSECA LM, 2020, SUSTAINABILITY-BASEL	86
BRATIANU C, 2020, KYBERNETES	53
BUTU A, 2020, INT J ENV RES PUB HE	49
KLIESTIK T, 2020, OECON COPERNIC	45
GHERGHINA SC, 2020, SUSTAINABILITY-BASEL	38
BRATIANU C, 2020, SUSTAINABILITY-BASEL	34
PELAU C, 2021, COMPUT HUM BEHAV	30
TARTAVULEA CV, 2020, AMFITEATRU ECON	29
RADULESCU CV, 2021, SUSTAINABILITY-BASEL	28
BRATIANU C, 2020, MANAG MARK-a	27
DAVIDESCU AA, 2020, SUSTAINABILITY-BASEL	25
SALARI M, 2020, J AIR TRANSP MANAG	25
VATAMANESCU EM, 2020, J KNOWL MANAG	25
DING LL, 2020, OCEAN COAST MANAGE	24
ARMEANU DS, 2021, RENEW SUST ENERG REV	22
SERBAN AC, 2020, IEEE ACCESS	22
GHERGHINA SC, 2020, INT J ENV RES PUB HE	21
VASILESCU MD, 2020, PLOS ONE	21
DING LL, 2020, J CLEAN PROD	21
**(b)**	
**Paper**	**Total Citations**
SORIANO JB, 2020, LANCET RESPIR MED	316
KLIESTIK T, 2020, ECON MANAG FINANCIAL MARK	98
HAILE LM, 2021, LANCET	89
KLIESTIK T, 2020, OECON COPERNIC	88
CEPOI CO, 2020, FINAN RES LETT	81
PELAU C, 2021, COMPUT HUM BEHAV	71
BUTU A, 2020, INT J ENVIRON RES PUBLIC HEALTH	62
GHERGHINA SC, 2020, SUSTAINABILITY	57
JAMES SL, 2020, INJURY PREV-a	49
BRATIANU C, 2020, KYBERNETES	43
DAVIDESCU AA, 2020, SUSTAINABILITY	42
TARTAVULEA CV, 2020, AMFITEATRU ECON	40
ANDREI JV, 2020, J BUS ECON MANAGE	32
SERBAN AC, 2020, IEEE ACCESS	32
ANDRONICEANU A, 2020, ADM MANAGE PUBLIC-a	31
BRATIANU C, 2020, SUSTAINABILITY	28
GHERGHINA C, 2020, INT J ENVIRON RES PUBLIC HEALTH	26
DING LL, 2020, J CLEAN PROD	26
ARMEANU DS, 2021, RENEWABLE SUST ENERGY REV	25
SALARI M, 2020, J AIR TRANSP MANAGE	25

## Data Availability

Data can be available upon request.

## References

[1] OECD (2020). OECD Economic Outlook.

[2] IMF (2020). World Economic Outlook: The Great Lockdown.

[3] Odone A., Salvati S., Bellini L., Bucci D., Capraro M., Gaetti G., Amerio A., Signorelli C. (2020). The runaway science: A bibliometric analysis of the COVID-19 scientific literature. Acta Biomater..

[4] Joshua V., Sivaprakasam S. (2020). Coronavirus: Bibliometric analysis of scientific publications from 1968 to 2020. Med. J. Islam. Repub. Iran.

[5] Hu B., Guo H., Zhou P. (2021). Characteristics of SARS-CoV-2 and COVID-19. Nat. Rev. Microbiol..

[6] Fan J., Gao Y., Zhao N., Dai R., Zhang H., Feng X., Shi G., Tian J., Chen C., Hambly B.D. (2020). Bibliometric Analysis on COVID-19: A Comparison of Research Between English and Chinese Studies. Front. Public Health.

[7] Elhawary H., Salimi A., Diab N., Smith L. (2020). Bibliometric Analysis of Early COVID-19 Research: The Top 50 Cited Papers. Infect. Dis. Res. Treat..

[8] Yang Y., Yang M., Yuan J., Wang F., Wang Z., Li J., Zhang M., Li X., Wei J., Peng L. (2020). Laboratory Diagnosis and Monitoring the Viral Shedding of SARS-CoV-2 Infection. Innovation.

[9] Mahi M., Mobin M.A., Habib M., Akter S. (2021). Knowledge Mapping of Pandemic and Epidemic Studies in Economics: Future Agenda for COVID-19 Research. Soc. Sci. Humanit. Open.

[10] Verma S., Gustafsson A. (2020). Investigating the emerging COVID-19 research trends in the field of business and management: A bibliometric analysis approach. J. Bus. Res..

[11] Aristovnik A., Keržič D., Ravšelj D., Tomaževič N., Umek L. (2020). Impacts of the COVID-19 Pandemic on Life of Higher Education Students: A Global Perspective. Sustainability.

[12] Hossain M.M. (2020). Current status of global research on novel coronavirus disease (COVID-19): A bibliometric analysis and knowledge mapping. F1000Research.

[13] Lee J.J., Haupt J.P. (2021). Scientific globalism during a global crisis: Research collaboration and open access publications on COVID-19. High. Educ..

[14] Raman R., Vinuesa R., Nedungadi P. (2021). Bibliometric Analysis of SARS, MERS, and COVID-19 Studies from India and Connection to Sustainable Development Goals. Sustainability.

[15] Duan D.Z., Xia Q.F. (2020). Evolution of scientific collaboration on COVID-19: A bibliometric analysis. Learn. Publ..

[16] Ahmed A. (2019). Bibliometric Analysis of Research Publications of Al-Jouf University, Saudi Arabia during the Year 2006–2017.

[17] Maharana R.K. (2013). A bibliometric analysis of the research output of Sambalpur University’s publication in ISI Web of Science during 2007–2011. Libr. Philos. Pract..

[18] Xianli W., Huchang L., Tang M., Ruxanda G., Smeureanu I. (2020). Global trends and characteristics of the publications in economic computation and economic cybernetics studies and research from 1969 to 2020 based on bibliometric analysis. Econ. Comput. Econ. Cybern. Stud. Res..

[19] Martínez-López F.J., Merigó J.M., Valenzuela-Fernández L., Nicolás C. (2018). Fifty years of the European Journal of Marketing: A bibliometric analysis. Eur. J. Mark..

[20] Maharana R.K., Das P. (2013). Research publication trend of Utkal University’s researchers indexed in Scopus during 2008 to 2012: A bibliometric analysis. Libr. Philos. Pract..

[21] Podsakoff P.M., MacKenzie S.B., Podsakoff N.P., Bachrach D.G. (2008). Scholarly Influence in the Field of Management: A Bibliometric Analysis of the Determinants of University and Author Impact in the Management Literature in the Past Quarter Century. J. Manag..

[22] Soytas R.B. (2021). A Bibliometric Analysis of Publications on COVID-19 and Older Adults. Ann. Geriatr. Med. Res..

[23] Gaviria-Marin M., Merigo J.M., Popa S. (2018). Twenty years of the Journal of Knowledge Management: A bibliometric analysis. J. Knowl. Manag..

[24] Siwach A.K., Parmar S. (2018). Research Contributions of CCS Haryana Agricultural University, Hisar: A Bibliometric Analysis. DESIDOC J. Libr. Inf. Technol..

[25] Belter C.W., Garcia P.J., Livinski A.A., Leon-Velarde F., Weymouth K.H., Glass R.I. (2019). The catalytic role of a research university and international partnerships in building research capacity in Peru: A bibliometric analysis. PLOS Negl. Trop. Dis..

[26] Zhou Y., Chen L. (2020). Twenty-Year Span of Global Coronavirus Research Trends: A Bibliometric Analysis. Int. J. Environ. Res. Public Health.

[27] Brodaus R.N. (1987). Early Approaches to Bibliometrics. J. Am. Soc. Inf. Sci..

[28] Pritchard A. (1969). Statistical Bibliography or Bibliometrics?. J. Doc..

[29] Wallin J.A. (2005). Bibliometric Methods: Pitfalls and Possibilities. Basic Clin. Pharmacol. Toxicol..

[30] Ramos-Rodríguez A.-R., Navarro J.R. (2004). Changes in the intellectual structure of strategic management research: A bibliometric study of theStrategic Management Journal, 1980–2000. Strat. Manag. J..

[31] Donthu N., Kumar S., Mukherjee D., Pandey N., Lim W.M. (2021). How to conduct a bibliometric analysis: An overview and guidelines. J. Bus. Res..

[32] Xu X., Chen X., Jia F., Brown S., Gong Y., Xu Y. (2018). Supply chain finance: A systematic literature review and bibliometric analysis. Int. J. Prod. Econ..

[33] Zupic I., Čater T. (2015). Bibliometric methods in management and organization. Organ. Res. Methods.

[34] Donthu N., Gustafsson A. (2020). Effects of COVID-19 on business and research. J. Bus. Res..

[35] Andersen N. (2019). Mapping the expatriate literature: A bibliometric review of the field from 1998 to 2017 and identification of current research fronts. Int. J. Hum. Resour. Manag..

[36] Mongeon P., Paul-Hus A. (2016). The journal coverage of Web of Science and Scopus: A comparative analysis. Scientometrics.

[37] López-Illescas C., de Moya-Anegón F., Moed H.F. (2008). Coverage and citation impact of oncological journals in the Web of Science and Scopus. J. Inf..

[38] Vera-Baceta M.-A., Thelwall M., Kousha K. (2019). Web of Science and Scopus language coverage. Scientometrics.

[39] Archambault É., Vignola-Gagné É., Côté G., Larivière V., Gingrasb Y. (2006). Benchmarking scientific output in the social sciences and humanities: The limits of existing databases. Scientometrics.

[40] Singh V.K., Singh P., Karmakar M., Leta J., Mayr P. (2021). The journal coverage of Web of Science, Scopus and Dimensions: A comparative analysis. Scientometrics.

[41] Martín-Martín A., Thelwall M., Orduna-Malea E., Delgado López-Cózar E. (2021). Google Scholar, Microsoft Academic, Scopus, Dimensions, Web of Science, and OpenCitations’ COCI: A multidisciplinary comparison of coverage via citations. Scientometrics.

[42] Vieira E.S., Gomes J.A.N.F. (2009). A comparison of Scopus and Web of Science for a typical university. Scientometrics.

[43] Aria M., Cuccurullo C. (2017). Bibliometrix: An R-tool for comprehensive science mapping analysis. J. Informetr..

[44] Feng Y., Zhu Q., Lai K.-H. (2017). Corporate social responsibility for supply chain management: A literature review and bibliometric analysis. J. Clean. Prod..

[45] Waltman L., Calero-Medina C., Kosten J., Noyons E.C., Tijssen R.J., van Eck N.J., Wouters P. (2012). The Leiden Ranking 2011/2012: Data collection, indicators, and interpretation. J. Am. Soc. Inf. Sci. Technol..

[46] Garg K.C., Tripathi H.K. (2018). Bibliometrics and scientometrics in India: An overview of studies during 1995–2014 Part II: Contents of the articles in terms of disciplines and their bibliometric aspects. Ann. Libr. Inf. Stud..

[47] Kessler M.M. (1963). Bibliographic coupling between scientific papers. Am. Doc..

[48] Small H. (1973). Co-citation in the scientific literature: A new measure of the relationship between two documents. J. Am. Soc. Inf. Sci..

[49] Callon M., Courtial J.P., Turner W.A., Bauin S. (1983). From translations to problematic networks: An introduction to co-word analysis. Soc. Sci. Inf..

[50] Martyn J. (1964). Bibliographic Coupling. J. Doc..

[51] Egghe L. (2006). Theory and practise of the g-index. Scientometrics.

[52] Costas R., Bordons M. (2008). Development of a thematic filter for the bibliometric delimitation on interdisciplinary area: The case Marine Science. Rev. Esp. Doc. Cient..

[53] Mougenot B., Doussoulin J.-P. (2022). Conceptual evolution of the bioeconomy: A bibliometric analysis. Environ. Dev. Sustain..

